# Claudin-7 promotes the epithelial – mesenchymal transition in human colorectal cancer

**DOI:** 10.18632/oncotarget.2858

**Published:** 2014-12-03

**Authors:** Rahel Philip, Sarah Heiler, Wei Mu, Markus W. Büchler, Margot Zöller, Florian Thuma

**Affiliations:** ^1^ Department of Tumor Cell Biology, University Hospital of Surgery, Heidelberg; ^2^ University Hospital of General Surgery, Heidelberg

**Keywords:** Colorectal cancer, claudin 7, EpCAM, cancer initiating cells, metastasis

## Abstract

In colorectal cancer (CoCa) EpCAM is frequently associated with claudin-7. There is evidence that tumor-promoting EpCAM activities are modulated by the association with claudin-7. To support this hypothesis, claudin-7 was knocked-down (kd) in HT29 and SW948 cells.

HT29-cld7^kd^ and SW948-cld7^kd^ cells display decreased anchorage-independent growth and the capacity for holoclone-, respectively, sphere-formation is reduced. Tumor growth is delayed and cld7^kd^ cells poorly metastasize. In line with this, migratory and invasive potential of cld7^kd^ clones is strongly impaired, migration being inhibited by anti-CD49c, but not anti-EpCAM, although motility is reduced in EpCAM siRNA-treated cells. This is due to claudin-7 recruiting EpCAM in glycolipid-enriched membrane fractions towards claudin-7-associated TACE and presenilin2, which cleave EpCAM. The cleaved intracellular domain, EpIC, promotes epithelial-mesenchymal transition (EMT)-associated transcription factor expression, which together with fibronectin and vimentin are reduced in claudin-7^kd^ cells. But, uptake of HT29^wt^ and SW948^wt^ exosomes by the claudin-7^kd^ lines sufficed for transcription factor upregulation and for restoring motility.

Thus, claudin-7 contributes to motility and invasion and is required for recruiting EpCAM towards TACE/presenilin2. EpIC generation further supports motility by promoting a shift towards EMT. Notably, EMT features of cld7-competent metastatic CoCa cells can be transferred via exosomes to poorly metastatic cells.

## INTRODUCTION

Colorectal cancer (CoCa) ranks on the 3^rd^ position of cancer incidence worldwide and accounts for the 4^th^ most common cancer death [1]. Though prognosis is improving with a 65% cure rate in high income countries, death rate still ranges between 20.1 for men and 12.2 for women / 100.000 persons in western countries [1-3]. Like for most cancer types, it is suggested that a small population of cancer initiating cells (CIC) [4] accounts for primary tumor growth as well as metastatic spread [5].

CIC are slowly progressing through the cell cycle [6], are highly radiation and drug resistant [7] and may use signaling pathways guiding the fate of embryonic and adult stem cells [8]. CIC can also be defined by a set of markers, which for CoCIC include CD44/CD44v6, EpCAM (EpC), Lgr5, CD133, CD166, Msi-1, CD29, CD24 [9], Tspan8 and claudin7 (cld7) [10]. The contribution of these markers to oncogenesis and/or tumor progression was, so far, unequivocally demonstrated only for CD44 [11,12]. But great efforts are undertaken to elaborate, whereby CIC account for tumor progression and therapy resistance, as it is hoped that attacking CIC offers more efficient therapeutics [13].

From the described CoCIC markers we are particularly interested in EpC, which interferes with E-cadherin mediated cell-cell adhesion via disrupting the link between β-catenin and F-actin [14]. EpC is also engaged in Wnt/β-catenin signaling [15], down-regulation of PKC [16] and regulation of MMP7 expression [17,18]. Furthermore, EpC cross-linking triggers TACE (TNFα converting enzyme) and PS2/NTF (presenilin 2 N-terminal fragment). The latter cleaves the intracellular domain of EpC, EpIC, which together with β-catenin, FHL2 (four-and-half-LIM-only) and Lef-1, relocates to the nucleus and initiates, besides others, c-myc, cyclinA and E [19,20], Oct4 and Nanog transcription [21]. EpIC also contributes to the process of epithelial-mesenchymal transition (EMT) by upregulation of vimentin, Snail, Slug and downregulation of E-cadherin [22]. We experienced that in colon and pancreatic cancer, EpC associates with cld7 [23], also described for hepatocyte progenitors [24].

Claudins, tight junction proteins [25], are partitioned into glycolipid-enriched membrane microdomains (GEM) upon palmitoylation, where they interact with scaffold proteins creating a platform for signal transduction and a linkage to the cytoskeleton [26]. Claudins are targets for several phosphokinases [27,28], cld phosphorylation prohibiting integration into tight junctions [29]. In line with this, a cld7 knockout (cld7^ko^) is lethal within 10 days after birth due to destruction of the intestine [30]. The authors speculate on the importance of a missing association with integrins and a striking upregulation of MMP3 contributing to gut destruction [30]. Notably, an EpC^ko^ also is associated with intestine destruction-promoted death within one week after birth, due to the missing association of EpC with cld7 [31].

Based on our experience in a rat pancreatic adenocarcinoma model, where the metastatic capacity was strongly impaired by an EpC knockdown (EpC^kd^) or a cld7^kd^ [10] we suggested that cld7 and EpC act as a tandem in tumor progression. To substantiate our hypothesis, cld7 was knocked down in 2 CoCa lines highly expressing EpC and cld7. We show that the EpC-cld7 association particularly contributes to EMT and that exosomes derived from EpC-cld7 competent CoCa suffice for EMT induction.

## RESULTS

EpC is known as a CoCIC marker [9], where we recently provided evidence in a rat tumor model that a concerted action of cld7 and EpC is required for tumor progression / metastasis formation [10]. Thus, we now asked, using human CoCa lines, for the underlying mechanism.

### The impact of cld7 on EpC and other potential CoCIC markers

**Figure 1:**
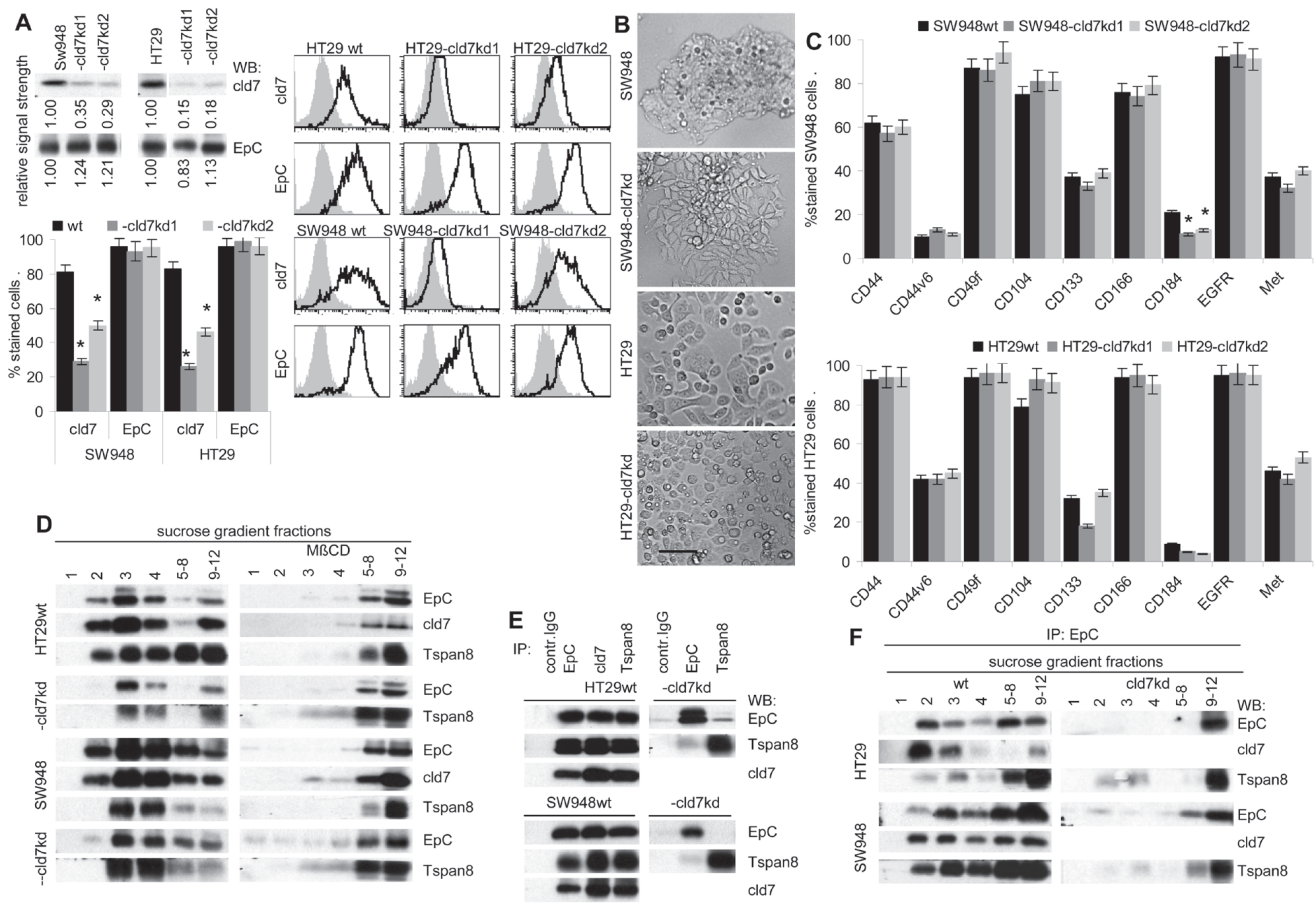
Characterization of cld7^kd^ colorectal cancer cells (A) WB and flow cytometry of SW948 and HT29 cells that were stably transfected with cld7 shRNA. Two clones of each line were selected for further experiments. WB and flow cytometry (mean % stained cells±SD, 3 assays and examples) of parental cells and two cld7^kd^ clones are shown. (B) Morphology of SW948^wt^ and HT29^wt^ and -cld7^kd^ cells (bar size: 25μm). (C) Expression of additional CoCIC markers in SW948^wt^ and HT29^wt^ and -cld7^kd^ clones (mean % stained cells±SD). (A,C): significant differences between wt and cld7^kd^ cells: *. (D) Sucrose density gradient fractionation of SW948^wt^ and HT29^wt^ and -cld7^kd^ lysates and WB of light density fractions 1-4 and pools of fractions 5-8 and 9-12 with anti-EpC, anti-cld7 and as control for a GEM-located molecule with anti-Tspan8. (E) Lysate of SW948^wt^ and HT29^wt^ and -cld7^kd^ cells were immunoprecipitated with control IgG, anti-EpCAM, anti-cld7 and anti-Tspan8. After SDS-PAGE separation, immunoprecipitates were blotted with anti-EpC, anti-cld7 and anti-Tspan8. (F) Immunoprecipitates as in (E) were separated by sucrose density gradient centrifugation. Light and pooled heavy fractions were separated by SDS-PAGE and blotted with anti-EpC, anti-cld7 and anti-Tspan8. A knockdown of cld7 in CoCa lines is not accompanied by downregulated expression of directly cld7-associated EpC and expression of additional CoCIC markers is not severely affected. However, only a minor part of EpC remains located in light density fractions (GEM).

To support the hypothesis that cld7 contributes to tumor progression-related activities of EpC, which frequently is associated with cld7 in CoCa, cld7 was knocked-down by shRNA transfection in the human CoCa lines HT29 and SW948, controlling common CIC features. The cld7^kd^ did not significantly affect EpC expression (Fig.1A). However, HT29-cld7^kd^ cells form less filipodia and lamellae than HT29^wt^ cells. The cell-cell contact of SW948-cld7^kd^ cells is loosened compared to SW948^wt^ cells and cells grow in multiple layers (Fig.1B). Besides a slight downregulation of CD184, which is weakly expressed by both lines, expression of additional CIC markers remained unimpaired (Fig.1C). Instead, the cld7^kd^ affected EpC recruitment in glycolipid-enriched, light sucrose density fractions (GEM) (Fig.1D). This is important, as GEM-located EpC does not form tetramers [23], tetramers being required for homophilic binding [14]. Co-immunoprecipitation of EpC with GEM-located proteins like the tetraspanin Tspan8 was strongly reduced (Fig.1E,1F). This suggested that in cld7^kd^ cells not only the membrane subdomain localization of EpC, but also the associations of EpC with additional membrane molecules are distorted (Fig.1E,1F).

Taken together, reduced cld7 expression hardly affects expression of additional CoCIC markers. However, the GEM-recruitment of EpC is strongly affected by reduced cld7 expression.

### Cld7 expression and CIC growth features of CoCa lines

**Figure 2:**
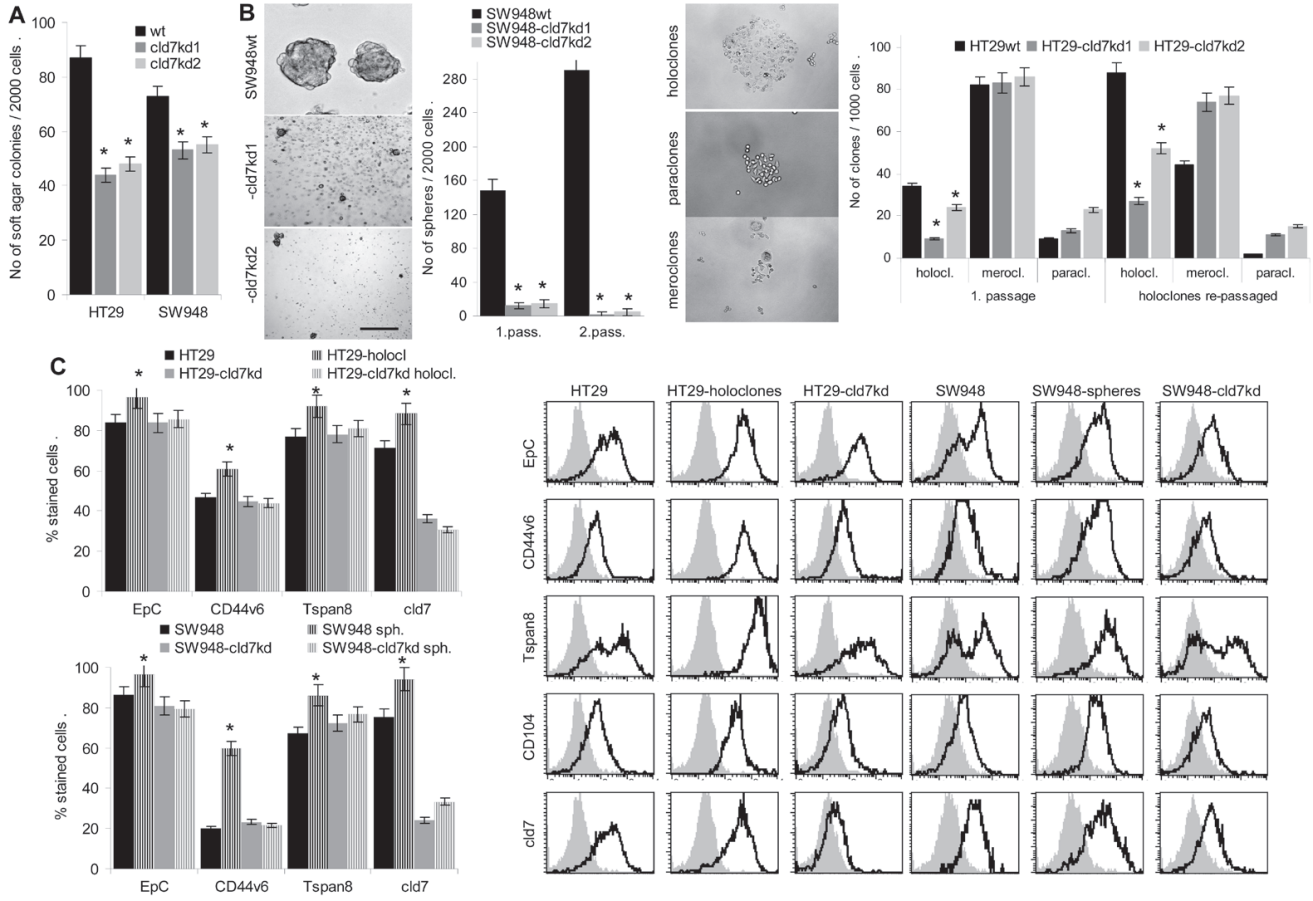

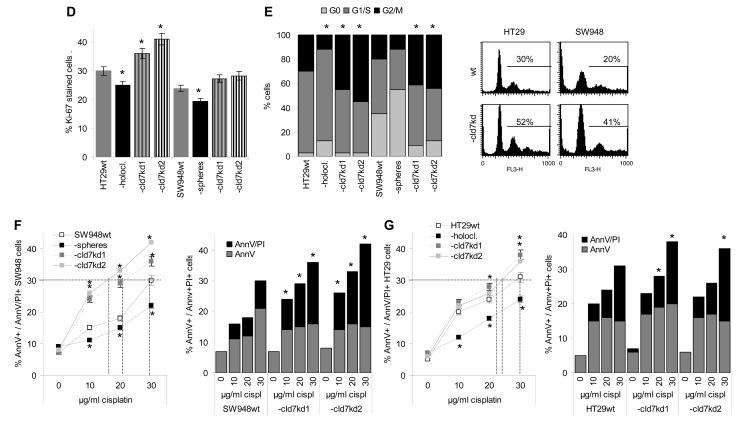
CIC growth features of CoCa-cld7^kd^ cells (A) No (mean±SD, 3 assays) of soft agar colonies of wt and cld7^kd^ cells; (B) SW948 cells were grown under spheroid growth condition and HT29 cells under condition for holoclone formation. Examples and mean numbers±SD (3 assays) of spheres (scale bar: 100μm) and holoclones, meroclones and paraclones (scale bar: 30μm) in a first round of cloning as well as after re-seeding 1^st^ passage spheres and holoclones are shown; (C) Expression of EpCAM, cld7, CD44v6 and Tspan8 in 1^st^ and 2^nd^ passage spheres and holoclones was evaluated by flow cytometry; % stained cells (±SD, 3 assays) and representative examples; (D) Ki-67 expression was evaluated by flow cytometry in wt, cld7^kd^ and holoclone- / sphere-derived cells; the mean percent±SD of stained cells is shown; (E) Cell cycle progression was evaluated by PI staining after starved cells had recovered for 2h in medium with FCS. The % of cells in G0, G1/S and G2/M and a representative example are shown. (F,G) SW948^wt^ and HT29^wt^, -cld7^kd^ and holoclone- / sphere-derived cells were cultured for 48h in the presence of titrated amounts of cisplatin. Apoptosis was determined after AnnV/PI staining by flow cytometry; % of cells stained for AnnV and AnnV plus PI (mean±SD, triplicates) and % of cells stained for AnnV or AnnV plus PI (mean of triplicates); (A-G) significant differences between wt versus cld7^kd^ and sphere- / holoclone-derived cells: *. Anchorage-independent growth and sphere / holoclone formation are impaired in cld7^kd^ cells, which is accompanied by a slightly increased proliferation rate, more rapid cycling and a distinct loss in apoptosis resistance.

CIC are characterized by anchorage-independent growth that was reduced by 50% in HT29-cld7^kd^ clones and by ~30% in SW948-cld7^kd^ clones (Fig.2A). Furthermore, the capacity of SW948-cld7^kd^ cells to grow as spheres was reduced over 10-fold compared to SW948^wt^ cells. When selecting first passage spheres and seeding them again under spheroid growth conditions, the percentage of spheres derived from SW948^wt^ 1^st^ passage spheres increased significantly. Instead, SW948-cld7^kd^ 1^st^ passage spheres did not gain in sphere forming capacity upon repassage. Similarly, the capacity of HT29^wt^ cells to grow as holoclones was reduced in HT29-cld7^kd^ clones and was only slightly increased upon repassage, whereas the number of repassaged HT29^wt^ 1^st^ passage holoclones was more than doubled upon repassage (Fig.2B). Notably, cld7 and EpC expression as well as expression of the CIC markers CD44v6 and Tspan8 was significantly increased in SW948 spheres and HT29 holoclones (Fig.2C).

The proliferation rate of HT29-cld7^kd^ and SW948-cld7^kd^ cells was slightly, but at least in HT29-cld7^kd^ cells significantly enhanced, whereas the proliferation rate of holoclones and spheres was slightly reduced (Fig.2D). Cell cycle progression was accelerated in HT29-cld7^kd^ and SW948-cld7^kd^ compared to wt cells and was reduced in HT29 holoclones (Fig.2E).

Thus, suggested CIC growth features are affected in cld7^kd^ CoCa lines. This accounts for sphere and holoclone formation and anchorage independent growth. As in spheres and holoclones high cld7 expression was seen in >90% of cells, spheres / holoclones of these two lines are well suited as internal controls for the cld7^kd^.

### Cld7 expression and CIC apoptosis-resistance of CoCa lines

Cisplatin resistance of SW948 spheres and HT29 holoclones was reduced as compared to wt cells. But cisplatin resistance of SW948-cld7^kd^ and, less pronounced, HT29-cld7^kd^ cells was impaired compared to wt cells. This is demonstrated for mitochondrial integrity, ^3^H-thymidine uptake (Suppl.Fig.1A-1D) and AnnV binding / PI uptake. While 25-30μg / ml cisplatin were required for a 30% death rate of SW948 and HT29, 15-20μg / ml cisplatin sufficed for a 30% death rate of SW948-cld7^kd^ and 20-22μg / ml for HT29-cld7^kd^ clones. Instead, only 22% of SW948 spheres and 24% of HT29 holoclones became apoptotic in the presence of 30μg/ml cisplatin (Fig.2F,2G). Evaluation of caspase activity in cisplatin-treated HT29^wt^ and SW948^wt^ and cld7^kd^ cells showed unaltered Casp8 expression; cleaved Casp9 and activated Casp3 were slightly upregulated in cld7^kd^ clones. Furthermore, PI3K, Akt and BAD phosphorylation was reduced. Accordingly, Bcl2 and BclXl (SW948-cld7^kd^) expression was low, but BAX expression remained largely unaltered in SW948-cld7^kd^ and HT29-cld7^kd^ cells. With respect to MDR expression, HT29 differed from SW948 cells, MDR was downregulated in HT29-cld7^kd^ cells, but low expression in SW948-cld7^kd^ cells remained unaltered (Suppl.Fig.1E,1F).

Cld7 upregulation is accompanied by slightly increased drug resistance and downregulation is accompanied by a minor loss in drug resistance that is promoted by effector caspase activation and reduced activation of the anti-apoptotic PI3K/Akt pathway.

### The impact of cld7 on motility and invasiveness

**Figure 3:**
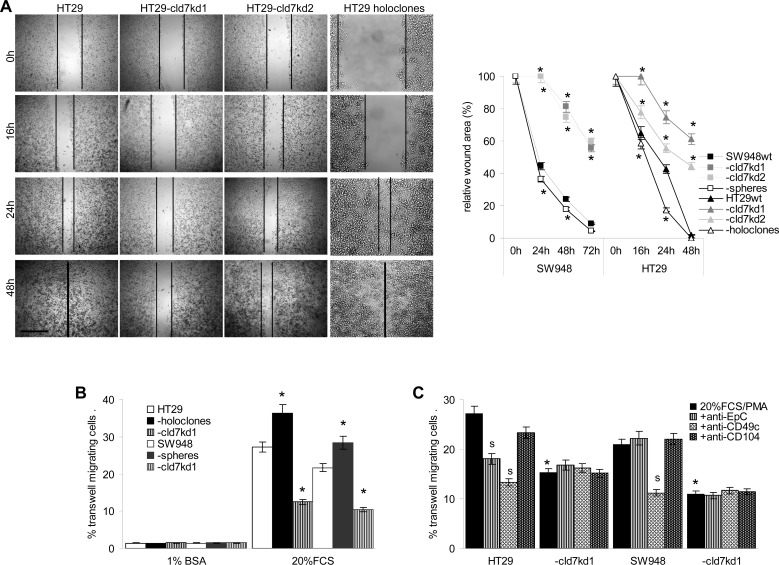

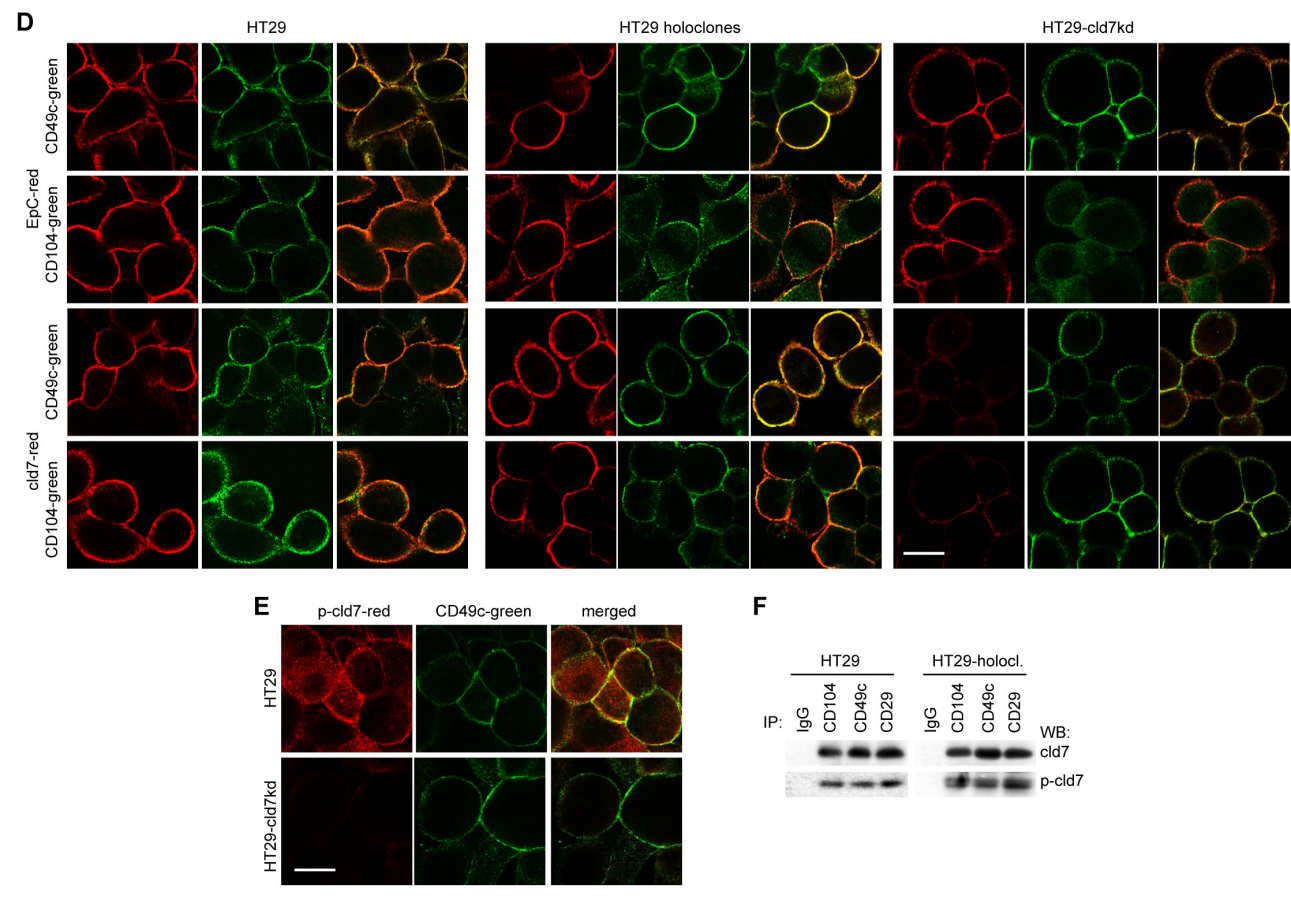
The impact of cld7 on CoCa cell migration (A) SW948^wt^ and HT29^wt^, -cld7^kd^ and holoclone- / sphere-derived cells were seeded in 24-well plates. When reaching subconfluence, the monolayer was scratched with a yellow pipette tip; wound healing was followed for 48h-72h; representative examples (HT29, scale bar: 250μm) and the wound area (mean±SD, triplicates) after 16h-72h; (B) transwell migration of SW948^wt^ and HT29^wt^, -cld7^kd^ and holoclone- / sphere-derived cells was evaluated in Boyden chambers after overnight incubation using RPMI with 20% FCS and 10^−8^M PMA in the lower chamber as stimulus; in (C) cells were preincubated with anti-EpC, anti-CD49c or anti-CD104. Cells at the lower membrane site were stained with crystal violet and lysed. The % migrating cells (mean±SD, triplicates) is shown; (A-C) significant differences between wt and cld7^kd^ cells: *; (C) significant antibody inhibition: s. (D) HT29^wt^, -cld7^kd^ and holoclone-derived cells were seeded on coverslides and were stained after overnight incubation in the presence of PMA with anti-cld7 or anti-EpC and anti-CD49c or anti-CD104. Single fluorescence and overlays of double staining are shown; scale bar: 10μm; (E) HT29^wt^ and –cld7^kd^ cells were stained with anti-CD49c and counterstained with anti-p-cld7; representative single fluorescence and overlays (scale bar: 10μm) are shown; (F) HT29^wt^ and holoclone-derived cells were PMA-treated and lysed. Lysates were precipitated with control IgG, anti-α3 or anti-β1. Precipitates were separated by SDS-PAGE and blotted with anti-cld7 and anti-p-cld7. Cld7 expression promotes motility by preferentially associating with CD49c / CD29. Integrin-associated cld7 is phosphorylated.

Metastasizing CIC are supposed to have high migratory potential. As demonstrated in an in vitro wound healing assay, a cld7^kd^ is accompanied by strongly reduced migratory activity, whereas motility of HT29 holoclone-derived cells is increased (Fig.3A,Suppl.Fig.2). Transwell migration confirmed the contribution of cld7 to tumor cell motility. Compared to wt cells, migration of cld7^kd^ cells was reduced, but holoclone-derived cells displayed significantly enhanced migratory activity (Fig.3B). The contribution of cld7 to transwell migration relies on its association with integrins rather than EpC, as migration was weakly or not inhibited by anti-EpC, but strongly inhibited by anti-CD49c. Reduced migration of cld7^kd^ cells was not affected by anti-CD49c (Fig.3C). Cld7-promoted motility was accompanied by strong colocalization and co-immunoprecipitation of EpC and cld7 with CD49c, CD29 and CD104 that was enhanced in holoclones and reduced in cld7^kd^ cells (Fig.3D,3F). Confocal microscopy and co-immunoprecipitation revealed that integrin-associated cld7 is phosphorylated (Fig.3E,3F).

**Figure 4:**
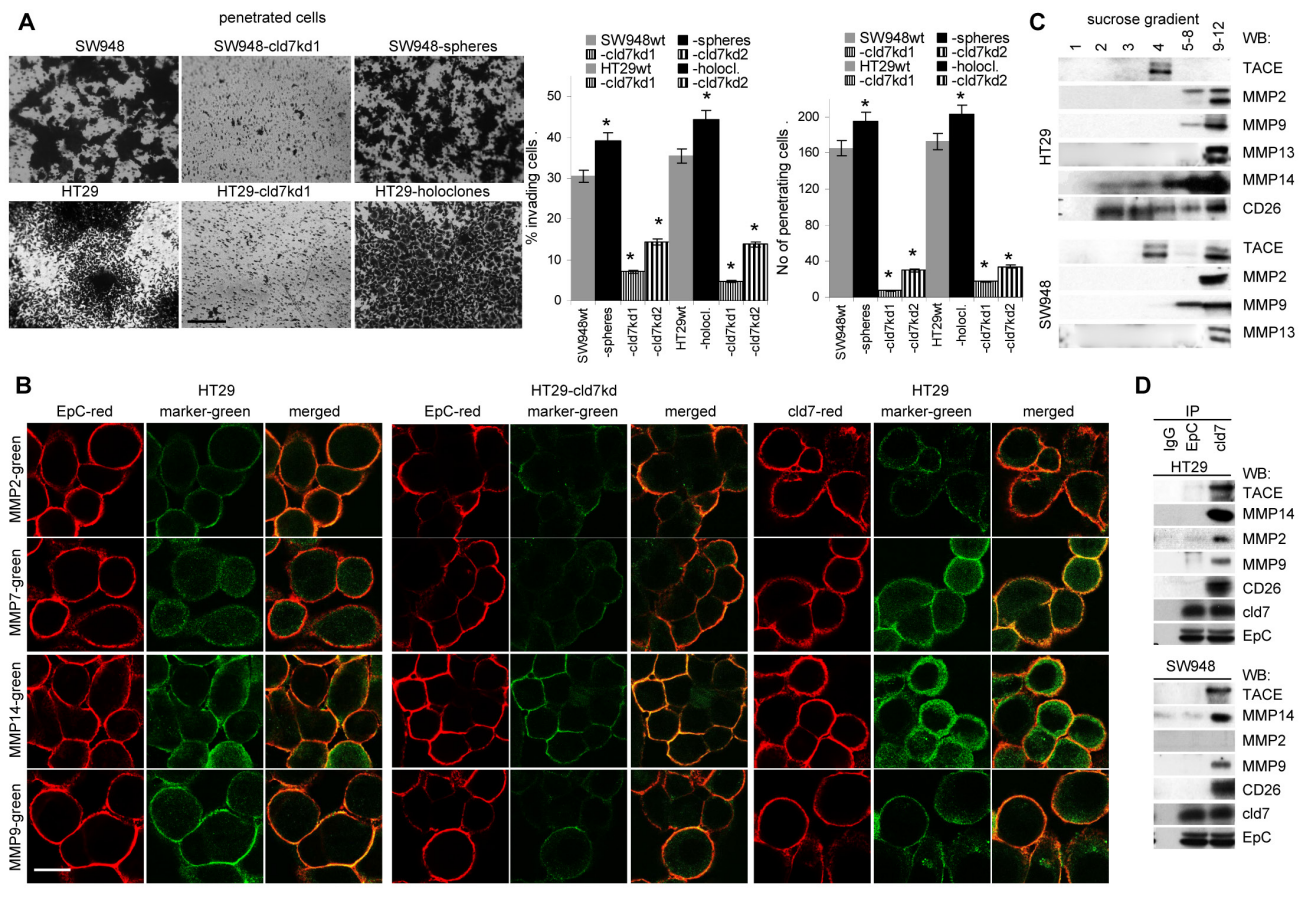
Cld7 and CoCa cell invasiveness (A) SW948^wt^ and HT29^wt^, -cld7^kd^ and sphere- / holoclone-derived cells were seeded on matrigel-coated inserts of a Boyden chamber; the lower chamber contained RPMI supplemented with 20% FCS. After overnight incubation, the matrigel block was stained with crystal violet and the cells that had invaded or penetrated the matrigel were counted; representative examples (scale bar: 250μm) and the number of invading and penetrating cells (mean±SD, triplicates) are shown; significant differences between wt versus cld7^kd^ or sphere- / holoclone-derived cells: *; (B) confocal microscopy of HT29 cells stained with anti-EpC or anti-cld7 and anti-MMP2, -MMP7, -MMP14 or –MMP9; single fluorescence and overlays of representative examples are shown (scale bar: 10μm). (C) Sucrose gradient fractions of lysates of HT29^wt^ and Sw948^wt^ and -cld7^kd^ cells were separated by SDS-PAGE and blotted with anti-TACE, -MMP2, -MMP9, -MMP13, -MMP14 and -CD26; (D) Lysates of HT29^wt^ and Sw948^wt^ and -cld7^kd^ cells were precipitated with control IgG, anti-EpC or anti-cld7. After SDS-PAGE separation, precipitates were blotted with anti-TACE, -MMP2, -MMP9, -MMP14 or -CD26. Cld7 contributes to invasiveness that might be promoted by proximity to MMP14 in GEM.

Finally, cld7 contributed to HT29 and SW948 invasiveness, which was enhanced in HT29 holoclone- and SW948 sphere-derived cells and was strongly decreased in the kd lines (Fig.4A). Invasiveness might be supported by the co-localization of cld7 with MMP14 and MMP7 and, weaker, but distinct colocalization with MMP2 and MMP9 (Fig.4B). TACE, MMP14 and CD26 are recovered in GEM (Fig.4C), suggesting a possible association with cld7. Indeed, TACE, MMP14 and CD26 co-immunoprecipitated with cld7, but not with EpC. Weak coimmunoprecipitation of MMP2 and MMP9 with cld7 under mild lysis conditions might be a sequel of MMP14 focalizing these MMPs at the plasma membrane (Fig.4D). Notably, MMP14 is not downregulated in cld7^kd^ cells. This also accounts for additional MMPs, the dipeptidases CD13 and CD26 as well as uPAR. Instead, MMP9 and MMP3 expression is slightly increased in the cld7^kd^ cells (Suppl.Fig.3).

Taken together, cld7 promotes tumor cell motility, which is accompanied by phosphorylated cld7 associating with CD49c and CD104, and supports invasiveness, which could be provoked by the colocalization of cld7 with MMP14 in GEM. MMP14 focalizes MMP2 and MMP9 close to the cell membrane and supports their activation [35,36], which contributes to invasiveness.

### Cld7 supports metastasis formation

**Figure 5:**
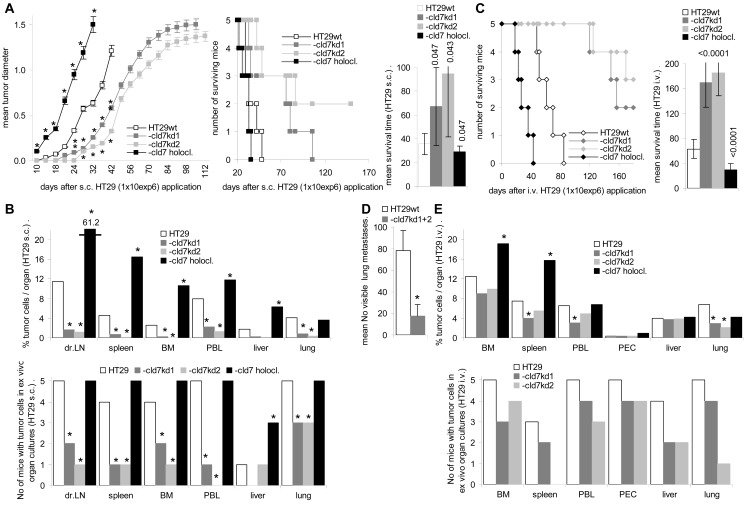
Metastatic settlement of HT29 cells in dependence on cld7 expression SCID mice (5/group) were treated with anti-asialoGM1, 24h in advance of receiving 1×10^6^ HT29 cells s.c. (A,B) or i.v. (C,D,E); (A) mean tumor diameter after s.c. injection of HT29^wt^, -cld7^kd^ or holoclone-derived cells, survival time and rate and mean survival time; significant differences in the tumor growth rate and the mean survival time depending on cld7 expression: * and p-values. (B) mice were sacrificed, when the subcutaneous tumor reached a mean diameter of 1.5cm, but latest after 210d. All mice were bled, and single cell suspensions were prepared from bone marrow, spleen, lymph nodes, lung and liver; the presence of tumor cells was evaluated by flow cytometry after staining with anti-EpC and anti-Tspan8; dispersed organs also were cultured for up to 4wk to observe tumor cell outgrowth. The mean percent of tumor cells in dispersed organs and the number of mice with tumor cell outgrowth in ex vivo organ cultures is shown; significant differences between mice bearing HT29^wt^, -cld7^kd^ or holoclone-derived cells: *. (C) Survival time, survival rate and mean survival time after i.v. tumor cell application; significant differences in the mean survival time depending on cld7 expression are indicated; (D) number of visible lung metastases (mean±SD) of mice that became sick after i.v. HT29 cell application; significant differences in the number of visible lung metastases: *; (E) All mice were bled, and single cell suspensions were prepared for flow cytometry and ex vivo cultures; the percent of tumor cells / organ and the number of mice with tumor cell outgrowth in ex vivo cultured organ suspension; significant differences between HT29^wt^, -cld7^kd^ and holoclone-derived tumor bearing mice: *. HT29-cld7^kd^ cell growth *in vivo* starts with delay and metastatic spread is impaired after s.c. and, less pronounced, i.v. application. HT29 holoclone-derived cells show a significantly accelerated growth rate and most efficiently settle and grow in draining lymph nodes after s.c. application.

Cld7-promoted motility and invasiveness supports metastasis formation. SCID mice received an s.c. or i.v. application of HT29 cells. Subcutaneous growth of HT29-cld7^kd^ cells started with delay and the survival time was significantly prolonged. Instead, the survival time of mice receiving holoclones was significantly shortened as compared to that of mice receiving wt cells (Fig.5A). Macroscopic metastases were seen in the draining LN of all 5 mice receiving holoclones and in 3 of 5 mice receiving HT29^wt^, but not in the draining LN of mice receiving cld7^kd^ cells. Nonetheless, as revealed by flow cytometry of dispersed organs after double staining with anti-EpC and anti-Tspan8, draining LN of HT29-cld7^kd^-bearing mice contained few tumor cells. Few HT29-cld7^kd^ cells were also recovered in the peripheral blood and very few in lung, liver, BM and spleen. All these organs contained a significantly higher number of HT29^wt^ cells. With exception of the lung, recovery was further increased in mice receiving HT29 holoclone-derived cells. In ex vivo cultures HT29^wt^ cells grew in draining LN, the peripheral blood and the lung of all 5 mice; HT29-cld7^kd^ cells grew only in draining LN, spleen, BM and peripheral blood of 1 or 2 mice. In lung cultures, HT29-cld7^kd^ cells were recovered in 3 of 5 mice. With exception of the liver (3 of 5 mice), HT29 holoclone-derived cells were recovered in the organs of all 5 mice (Fig.5B). After i.v. tumor cell application, the survival time of HT29-cld7^kd^ bearing mice was significantly prolonged and 5 of the 10 mice receiving HT29-cld7^kd^ cells were still healthy 210d after tumor cell application. Mice that were sacrificed as they started to loose weight, showed lung metastases with the exception of one HT29-cld7^kd^ bearing mouse. HT29^wt^ bearing mice showed around 80 metastatic nodules, the 5 HT29-cld7^kd^ bearing mice that became sick showed 0-25 metastatic nodules (Fig.5C,5D). The tumor-load in the BM did not significantly differ between mice bearing wt or cld7^kd^ tumors, but was increased in mice receiving HT29 holoclone-derived cells. The tumor load in the peripheral blood, the spleen and the lung was reduced in cld7^kd^ bearing animals. Ex vivo outgrowth of tumor cells from dispersed organs confirmed that HT29-cld7^kd^ cells hardly settled and/or survived in liver and lung (Fig.5E).

Thus, cld7 promotes settlement in metastatic organs after s.c. and i.v. tumor cell application. In line with the strong impact on motility, these findings point towards cld7 being engaged in the transition from the sessile towards the motile state (EMT).

### EMT gene expression in cld7^kd^ cells

**Figure 6:**
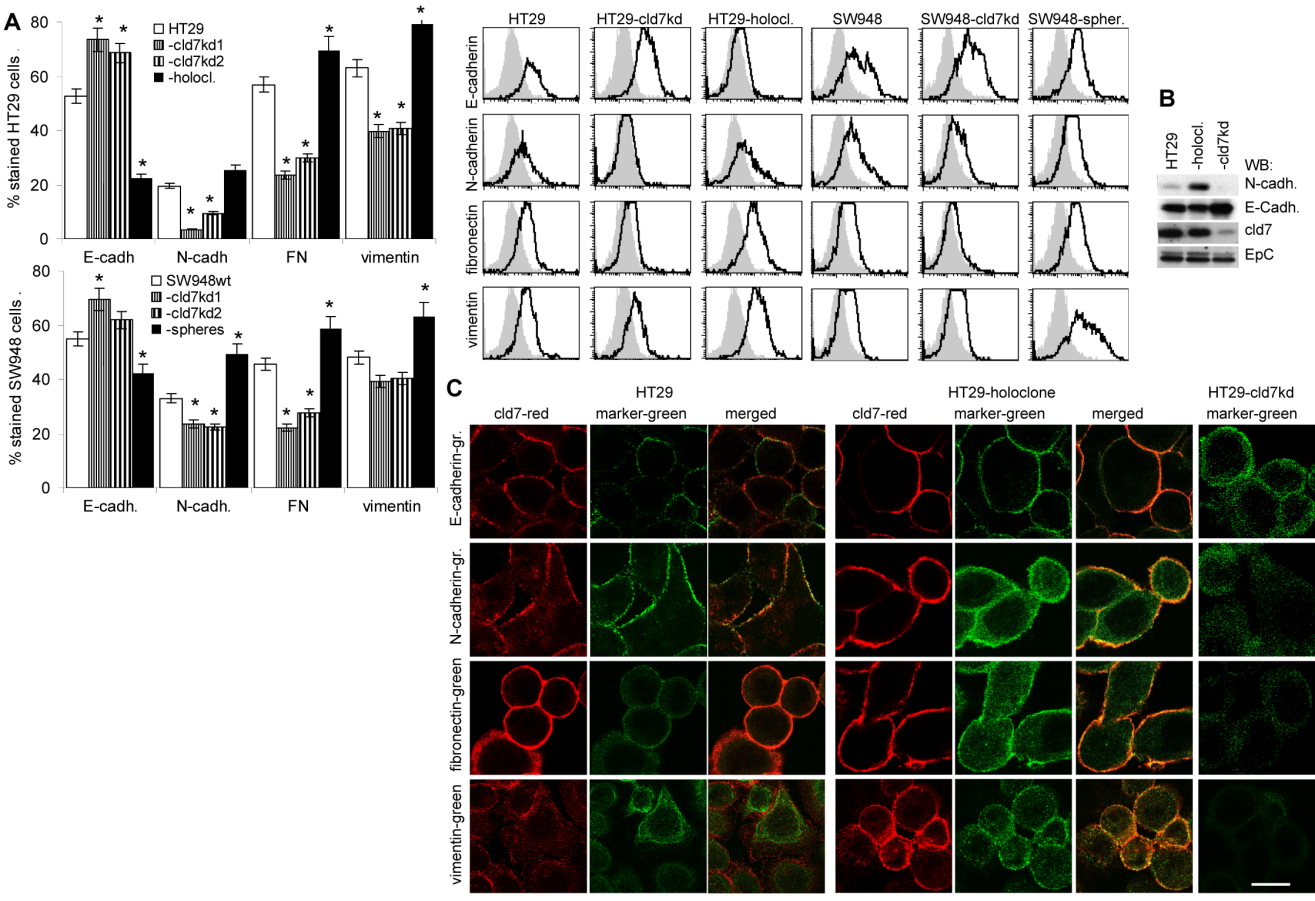

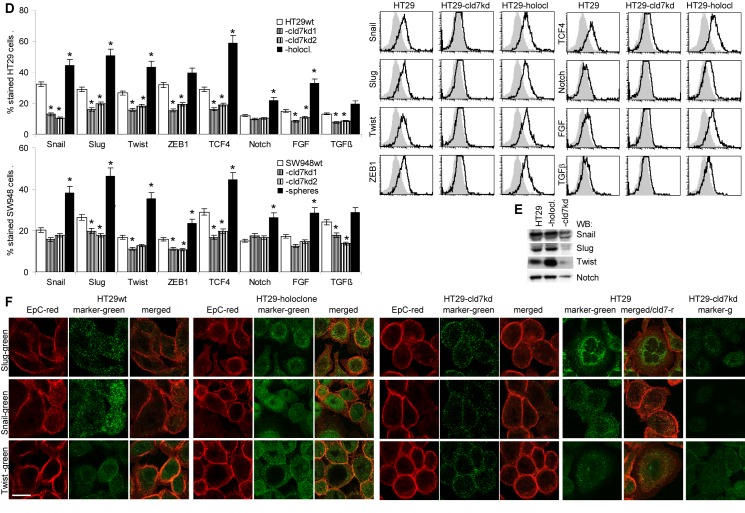
Expression of mesenchymal markers and EMT transcription factors in dependence on cld7 expression Expression of E-cadherin, N-cadherin, FN and vimentin was evaluated by (A) flow cytometry, the percent stained cells (mean±SD, 3 assays) and representative examples are shown; significant differences between wt, cld7^kd^ and holoclone- / sphere-derived cells: *; (B) WB and (C) confocal microscopy of HT29^wt^, -cld7^kd^ and holoclone-derived cells stained with anti- E-cadherin, -N-cadherin, -FN and -vimentin and counterstained with anti-cld7. Where indicated, single fluorescence staining and overlays of red and green fluorescence or green fluorescence are shown (scale bar: 10μm); (D) Flow cytometry analysis of transcription factors and cytokines supporting mesenchymal gene expression; the % stained cells (mean±SD, three assays) and representative examples are shown, significant differences between wt versus cld7^kd^ or holoclone- / sphere-derived cells: *; (E) WB of lysates as above, blotted with anti-Snail, -Slug, -Twist and -Notch; (F) Confocal microscopy of HT29^wt^, -cld7^kd^ and holoclone-derived cells stained with anti-EpC or anti-cld7 and/or anti-Slug, -Snail and -Twist; single fluorescence and overlays of red and green fluorescence or green fluorescence are shown (scale bar: 10μm). Cld7 expression supports the expression of mesenchymal proteins, which is accompanied by higher expression of transcription factors and cytokines supporting EMT protein expression.

Searching for EMT-related protein expression in holoclone-derived, wt and kd cells showed reduction of FN, N-cadherin and vimentin and upregulation of E-cadherin expression in cld7^kd^ compared to wt cells and an opposite regulation in HT29 holoclone-derived cells, which was confirmed for E-cadherin and N-cadherin by WB. Similar findings accounted for SW948^wt^ versus SW498-cld7^kd^ and -sphere-derived cells (Fig.6A,6B). As demonstrated for HT29^wt^ and HT29 holoclone-derived cells, FN and N-cadherin colocalize with cld7 in the plasma membrane, whereas vimentin is organized in the submembrane region. The reduction in vimentin, fibronectin and N-cadherin expression in cld7^kd^ cells is accompanied by redistribution with predominantly cytoplasmic localization. E-cadherin localization remained unaltered (Fig.6C).

The suggested engagement of cld7 in EMT prompted us to search for expression of factors known to contribute to EMT. Flow cytometry and WB showed upregulated expression of the transcription factors Snail, Slug, Twist, ZEB1, TCF4 and Notch in holoclones, but downregulation, though mostly to a minor degree in cld7^kd^ cells. FGF and TGFβ that support EMT protein expression [37,38] were downregulated in cld7^kd^ cells with a stronger impact of cld7 on FGF expression in HT29 than SW948 cells (Fig.6D,6E). Confocal microscopy confirmed downregulated expression of Slug, Snail and Twist in HT29-cld7^kd^ cells and upregulation in HT29 holoclones (Fig.6F).

Thus, a cld7^kd^ affected EMT gene expression. A possible explanation could rely on cld7 guiding EpC into GEM, where it becomes susceptible to digestion by TACE and subsequently by presenilin2, EpIC acting as a cotranscription factor besides others in cooperation with β-catenin [19-22].

### Cld7, EpIC and EMT

**Figure 7:**
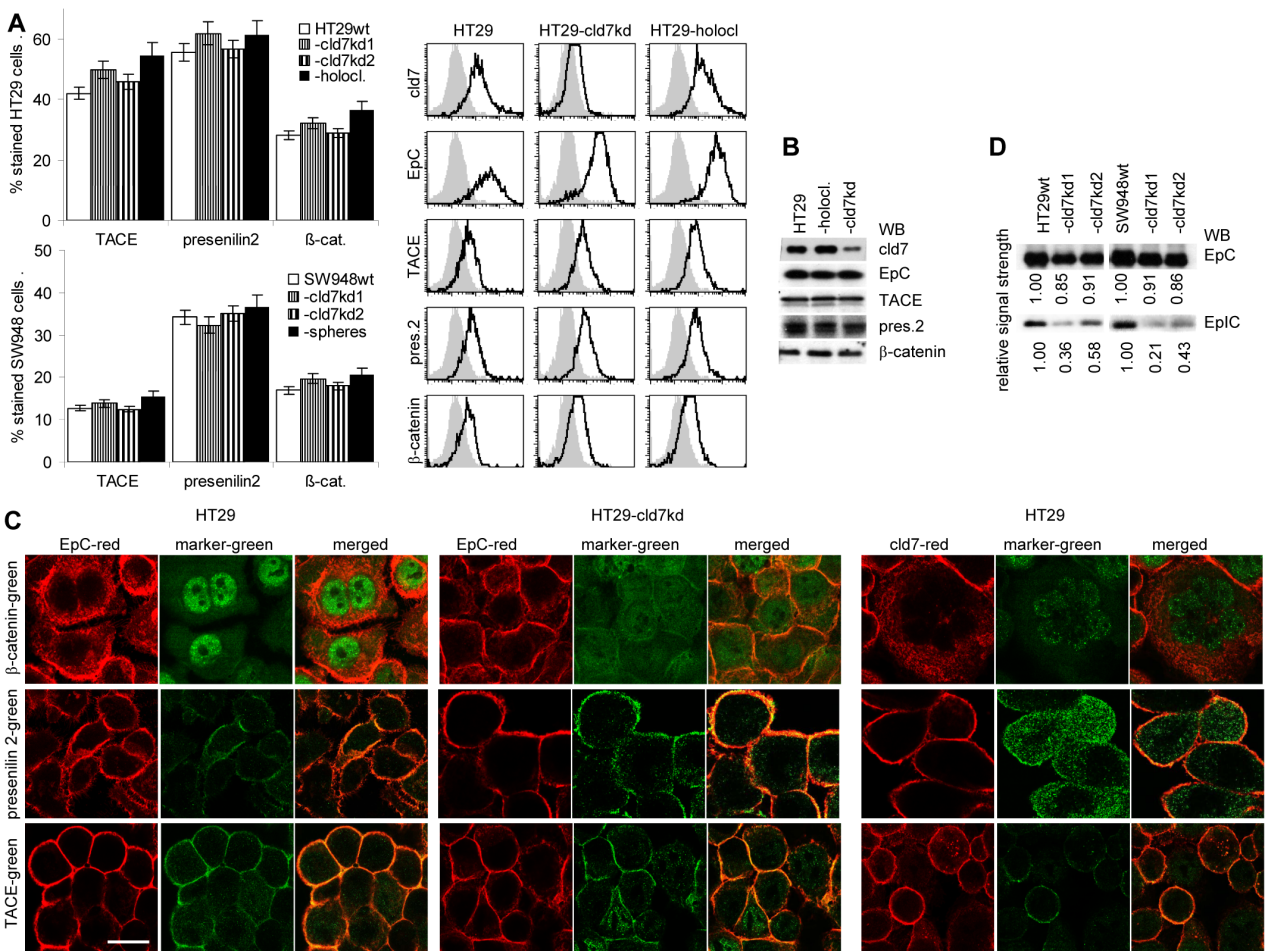

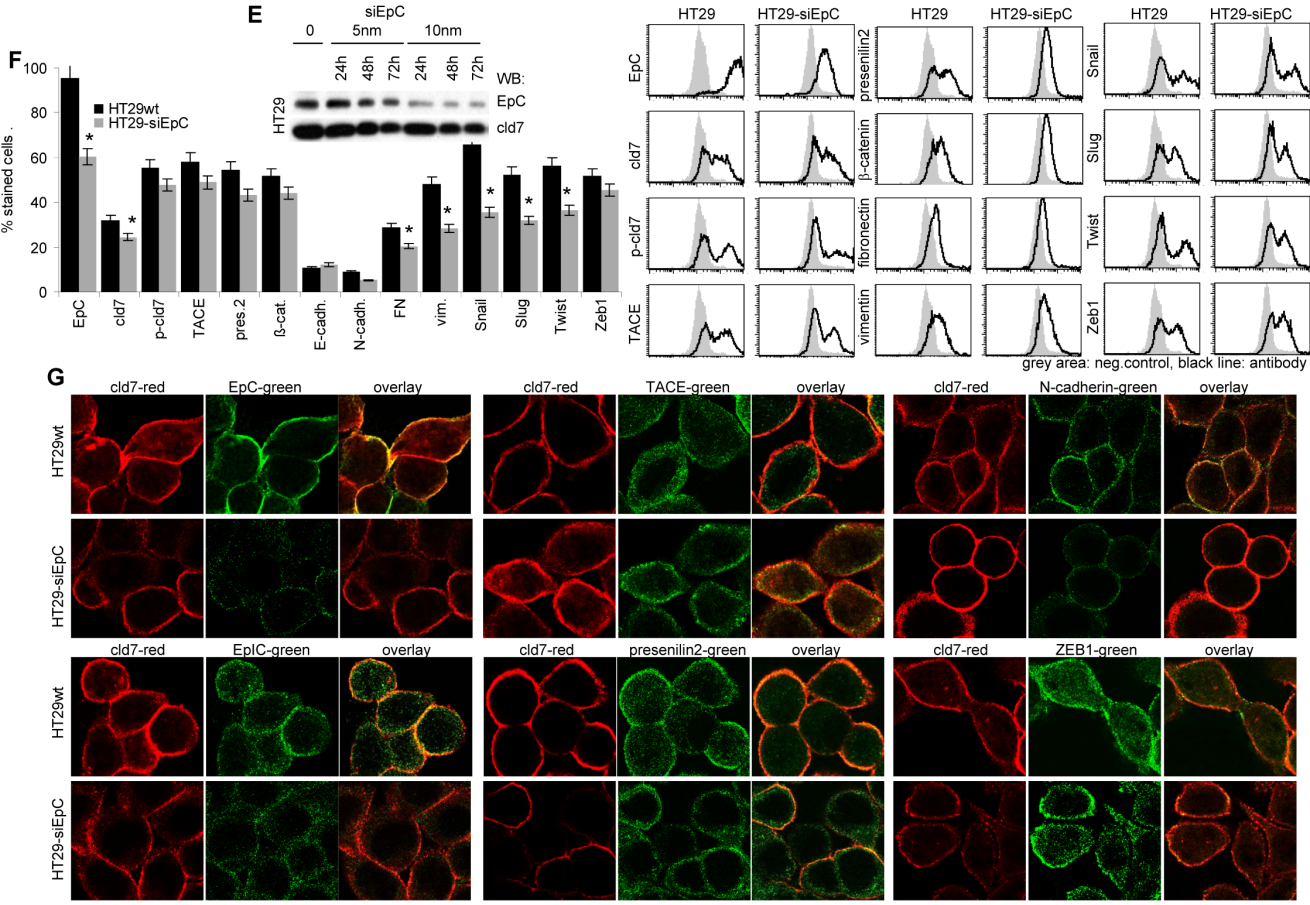
Cld7 expression and EpIC generation (A) Flow cytometry analysis of TACE, presenilin2 and β-catenin expression in HT29^wt^ and SW948^wt^, -cld7^kd^ and holoclone- / sphere-derived cells; the percent stained cells (mean±SD, three assays) and representative examples are shown; (B) WB of HT29wt, -cld7^kd^ and holoclone-derived lysates with the indicated antibodies; (C) confocal microscopy of β-catenin, presenilin2 and TACE staining and counterstaining with anti-EpC or -cld7 (single fluorescence and overlays of red and green fluorescence); scale bar: 10μm; (D) WB for EpC and EpIC in HT29^wt^, SW948^wt^ and -cld7^kd^ cells; (E) HT29 were transfected with titrated doses of EpC siRNA (Suppl.Table1). Downregulation of EpC expression was evaluated by WB after 24h, 48h and 72h. According to the expression profile, 10nm EpC siRNA were used and cells were tested after 48h. (F) EpC was transiently kd in HT29 and SW948 cells; EpC, TACE, presenilin, β-catenin and EMT gene expression was evaluated by flow cytometry (mean±SD, triplicates and representative examples); (G) confocal microscopy of single fluorescence staining and overlays of EpC, EpIC, TACE, presenilin2, N-cadherin and Zeb1 with cld7 (scale bar: 10μm). TACE, presenilin and β-catenin expression is not affected by a cld7^kd^. However, EpIC generation is severely impaired and Snail, Slug and Twist expression is reduced. This also accounts for a transient EpC^kd^.

TACE, presenilin and β-catenin expression is not significantly altered in HT29 holoclone- and SW948 sphere-derived cells compared to -cld7^kd^ and wt cells (Fig.7A,7B). However, in cld7^kd^ cells the intracellular distribution of β-catenin appears affected with a preferential submembrane localization (Fig.7C). Importantly, EpIC recovery is strikingly reduced (Fig7D). To confirm that the transitional state of CoCa cells requires EpIC and that cld7 provides one of the initial triggers for EpIC generation, we searched for mesenchymal gene expression in PMA-stimulated cld7^wt^ and cld7^kd^ cells with a transient EpC^kd^ (Fig.7E). Indeed, a transient EpC^kd^ was accompanied by downregulation of FN, vimentin, N-cadherin, Snail, Slug, Twist and ZEB1 expression (Fig.7F,7G).

These findings indicating a major contribution of cld7 to EMT via supporting the generation of EpIC, we finally asked, whether EMT can be transferred from metastasizing to poorly metastasizing cells via exosomes.

### Exosomes and EMT

**Figure 8:**
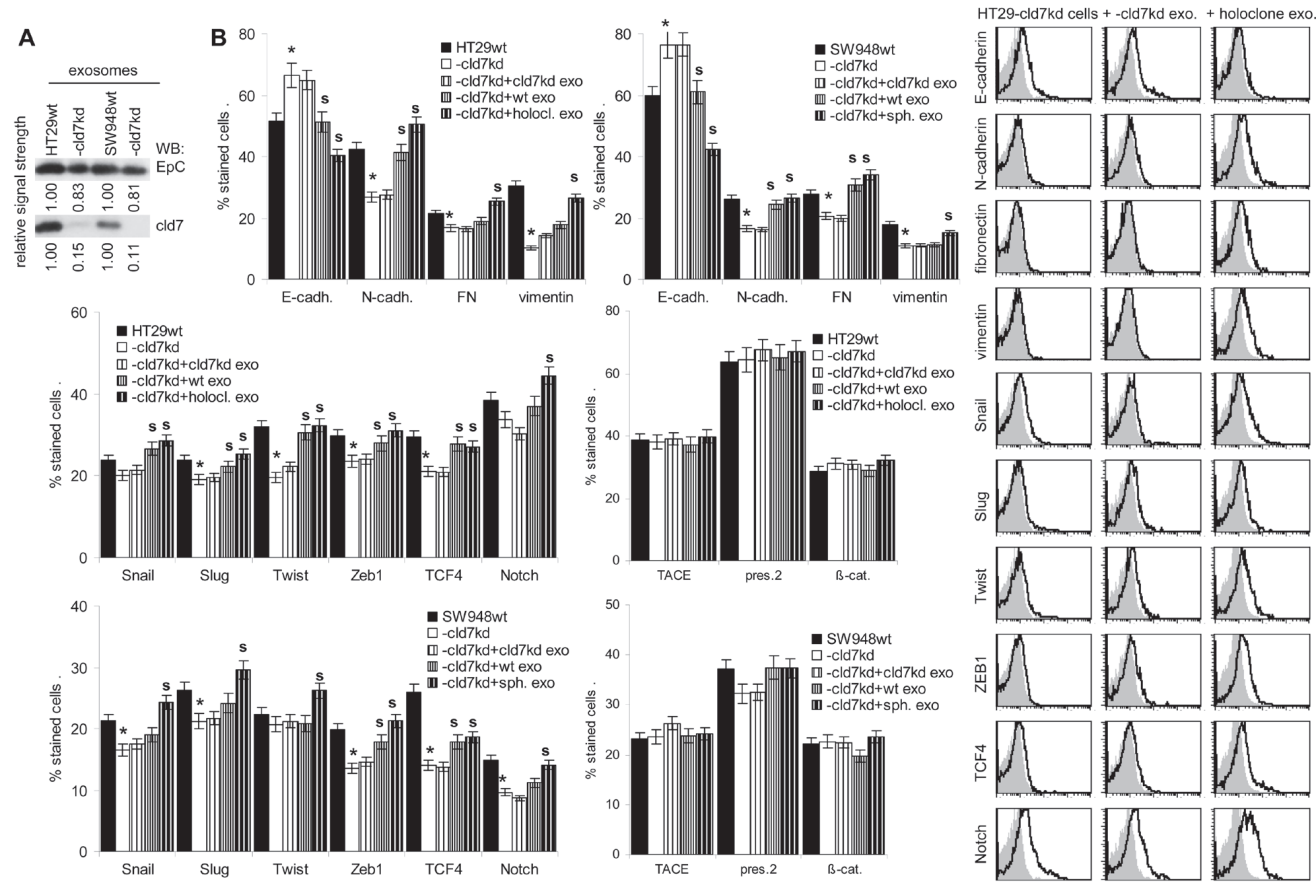

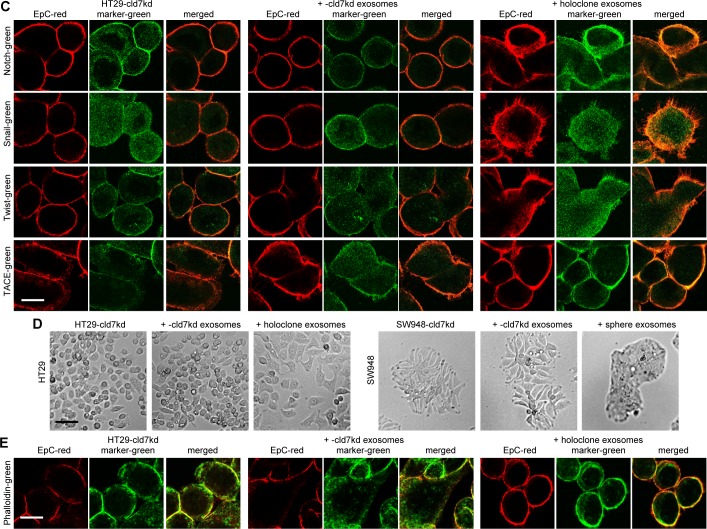

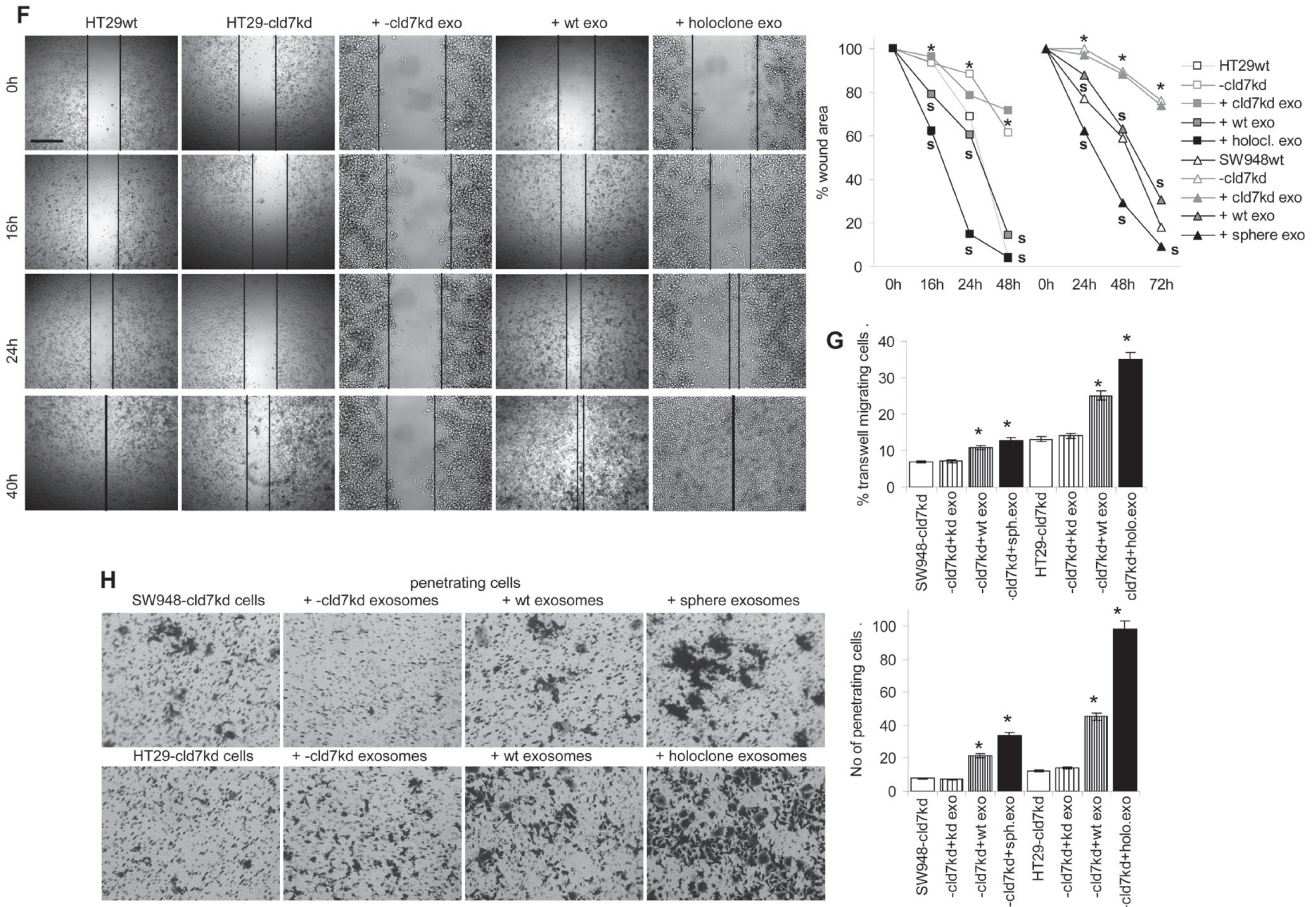
EMT induction by exosomes (A) WB of EpC and cld7 expression in HT29^wt^ and SW948^wt^ and -cld7^kd^ exosomes. (B-F) HT29-cld7^kd^ and SW948-cld7^kd^ cells were cultured for 48h with 10μg/ml exosomes derived from HT29^wt^ and SW948^wt^, -cld7^kd^ or sphere- / holoclone-derived cells. (B) Flow cytometry analysis of cld7, EpC and EMT gene expression; the % stained cells (mean±SD, triplicates) and representative examples are shown; significant differences between wt, cld7^kd^ and holoclone- / sphere-derived cells: *; (C) confocal microscopy of EMT gene expression in HT29-cld7^kd^ cells cocultured with cld7^kd^ or holoclone-derived exosomes; single fluorescence and overlays with anti-EpC are shown (scale bar: 10μm); (D) light microscopy of HT29-cld7^kd^ and SW948-cld7^kd^ cells after coculture with cld7^kd^ or holoclone- / sphere-derived exosomes (scale bar: 25μm); (E) confocal microscopy of HT29-cld7^kd^ cells cultured with/without cld7^kd^ or holoclone-derived exosomes; EpC (red), phalloidin-FITC and overlays are shown (scale bar: 10μm); (F) HT29-cld7^kd^ and SW948-cld7^kd^ cells were seeded in 24w plates; subconfluent cultures were scratched with a pipette tip. Wound healing was observed for 40h or 72h in the presence of medium with exosome-depleted FCS, or with exosomes from wt, cld7^kd^ or holoclone / sphere-derived cells; representative examples (scale bar: 250μm) and the wound area (mean±SD, triplicates) are shown; significant differences between wt versus cld7^kd^ cells: *, significant differences by coculture with wt, cld7^kd^ and holoclone- / sphere-derived exosomes: s. (G) HT29-cld7^kd^ and SW948-cld7^kd^ cells were seeded in the upper part of a Boyden chamber. The lower chamber contained 20% exosome-depleted FCS with/without 10μg/ml cld7^kd^, wt or holoclone- / sphere-derived exosomes; the percent migrating cells is shown; significant differences in the presence of exosomes: *; (H) SW948-cld7^kd^ and HT29-cld7^kd^ cells were seeded on matrigel-coated inserts of a Boyden chamber; the lower chamber contained RPMI supplemented with 20% exosome-depleted FCS with/without 10μg/ml cld7^kd^, wt or holoclone- / sphere-derived exosomes. After overnight incubation, the matrigel block was stained with crystal violet and the cells that had invaded or penetrated the matrigel were counted; representative examples (scale bar: 250μm) and the number of invading and penetrating cells (mean±SD, triplicates) are shown; significant differences in the presence of cld7^kd^, wt, or sphere- / holoclone-derived exosomes: *. Exosomes from cld7 competent cells suffice for upregulated expression of mesenchymal markers and genes in cld7^kd^ cells, which is accompanied by changes in cell shape as well as increased motility and invasiveness.

EpC and cld7 are recovered at a high level in exosomes. EpC expression was slightly reduced in exosomes from cld7^kd^ cells (Fig.8A), which fits the requirement of cld7 for the recruitment of EpC towards internalization prone GEM [10]. When HT29-cld7^kd^ and SW948-cld7^kd^ cells were cultured with exosomes derived from HT29^wt^ and SW948^wt^ cells, expression of N-cadherin, vimentin and FN (only SW948-cld7^kd^ cells) as well as of Snail, Twist, ZEB1 and TCF4 became upregulated, though more pronounced in HT29-cld7^kd^ than SW948-cld7^kd^ cells. Upregulated expression became more pronounced after coculture of cld7^kd^ cells with sphere- or holoclone-derived exosomes. This accounted particularly for vimentin and Notch. Instead, none of these effects where seen, when cld7^kd^ cells were cocultured with cld7^kd^ exosomes (Fig.8B), which was confirmed by confocal microscopy (Fig.8C). Furthermore, upon coculture with exosomes derived from holoclones or spheres the round / epitheloid shape of HT29-cld7^kd^ cells changed towards fibroid and SW948-cld7^kd^ cells again formed most tightly packed clusters (Fig.8D). Changes in cell shape were accompanied by reorganization of the actin cytoskeleton with actin bundle formation (Fig8E). Finally, HT29- and SW498-cld7^kd^ cells treated with wt exosomes regained full motility. Wound closure was further accelerated by coculture with exosomes from holoclones or spheres. Coculture with exosomes from cld7^kd^ cells exerted no effect (Fig.8F,Suppl.Fig.4). Treatment with wt or holoclone- / sphere-derived exosome, but not with cld7^kd^-derived exosomes also restored transwell migration (Fig.8G) and matrigel invasion, the efficacy of holoclone- / sphere-derived exosomes exceeding that of wt exosomes (Fig.8H). Thus, cld7-competent exosomes from metastasizing tumor cells suffice to initiate EMT in poorly metastasizing cld7^kd^ cells.

### spheres or holoclones from

In brief, cld7 downregulation in CoCa is accompanied by reduced anchorage-independent growth, sphere or holoclone formation, slightly reduced apoptosis resistance and a striking loss in motility. This corresponds to poor metastasis formation associated with reduced EMT. These phenomena are due to cld7 promoting motility by associating with integrins and by cld7 initiating the generation of EpIC, which contributes to EMT gene expression.

## DISCUSSION

EpC is known as a CIC marker in colorectal cancer, where we suggested that a contribution of EpC to tumor progression essentially depends on cooperativity with cld7 [9,23]. In line with our hypothesis, a cld7^kd^ as well as an EpC^kd^ of a rat pancreatic cancer line showed a striking loss in metastasis formation, where cld7 appeared to be the driving force [10]. Building on these findings, we here confirmed by a cld7^kd^ in two human colorectal cancer lines the general validity for the cld7-EpC cooperation being required for tumor progression. We suggest that cld7-promoted EpIC generation strongly supports the shift towards EMT.

### Impact of cld7 on CoCIC markers

The cld7^kd^ affected the cell shape, most pronounced of SW948 cells, where the extremely tight cell-cell contacts became weakened. HT29 cells shifted from a fibroid towards a more round shape. However, neither EpC nor expression of additional CoCIC markers was seriously affected. Instead, the recruitment of EpC in GEM was distorted and co-immunoprecipitation with the GEM-located CIC marker Tspan8 [39] was strikingly reduced. From there we conclude that cld7 does not significantly influence transcription / translation of EpC or additional CoCIC markers, but affects the distribution of EpC in the cell membrane.

### Cld7 influences stem cell features of CoCIC

Cld7^kd^ cells displayed a strongly reduced capacity for anchorage-independent growth, sphere (SW948) or holoclone (HT29) formation, whereas proliferation and cell cycle progression were slightly accelerated. It is tempting to speculate that the reduced activation of β-catenin (see below) accounts, at least in part, for the loss of CIC growth characteristics. The finding is in line with the Wnt/β-catenin pathway regulating maintenance of CoCa spheres [40]. Cld7 also supported apoptosis resistance, which became stronger in SW948 spheres than wt cells, whereas it was weakened in SW948-cld7^kd^ cells, which showed impaired activation of the PI3K/Akt pathway. Instead, high apoptosis resistance of HT29 cells was only weakly affected by the cld7^kd^. One possible explanation could be the distinct MDR gene expression, which is regulated by the CoCIC marker CD44v6 [41]. As CD44v6 also associates with cld7 in GEM [42,43], we suggest that the impact of a cld7^kd^ on apoptosis resistance, including the reduced activation of the PI3K/Akt pathway in cld7^kd^ lines is indirect and mediated by cld7-associated CD44v6 [44] such that a higher level of CD44v6 and MDR may compensate for reduced cld7 expression.

In brief, a cld7^kd^ hardly affected proliferation and had no strong impact on drug resistance. Instead, anchorage-independent growth was strongly impaired, which points towards cld7 being engaged in selective CIC activities.

### Cld7, motility, invasion and metastasis

Both HT29-cld7^kd^ and SW948-cld7^kd^ cells display strongly reduced motility. In line with this finding, recovery of migrating tumor cells in the peripheral blood was impaired after s.c. HT29-cld7^kd^ cell application. In a gastric cancer line and ovarian cancer, too, overexpression of cld7 was found to be associated with increased motility and invasiveness [45,46], although the opposite was described for lung cancer [47]. Cell migration *in vitro* was inhibited by anti-CD49c, but not in cld7^kd^ cells. Fittingly, the strong co-localization of EpC, cld7 and CD49c in the cell membrane is reduced in cld7^kd^ cells. Also, CD49c colocalization and co-immunoprecipitation with cld7 requires cld7 phosphorylation. In cld7^ko^ mice, expression of CD49b is significantly impaired and CD49b localization is distorted [30]. The finding suggests a major contribution of the cld7-integrin cooperation in promoting motility, which assumption is supported by cld7 associating with phosphorylated ezrin [9].

Cld7^kd^ cells are also characterized by strongly reduced matrigel invasion and after s.c. HT29-cld7^kd^ cell application only very few tumor cells are recovered from the lung; after i.v. application only 50% of HT29-cld7^kd^-bearing mice developed visible lung metastasis compared to 100% in HT29^wt^-bearing mice. A cld7^ko^ was reported to be accompanied by a strong upregulation of MMP3 [30]. However, in the cld7^kd^ tumor lines the effect was comparably weak and expression of other MMPs, two dipeptidases and uPAR was not consistently affected by either cld7 overexpression in spheres / holoclones or by low level expression in cld7^kd^ lines. Instead, MMP14 colocalizes and associates with cld7 in GEM fractions. MMP14 focalizes and supports activation of non-membrane-bound MMPs close to the cell membrane [35,36] and this could well explain higher protease activity in cld7^wt^ compared to cld7^kd^ cells.

Taken together, the association of phosphorylated cld7 with integrins, which is accompanied by FAK activation and ezrin association, contributes to tumor cell motility and the association with MMP14 in GEM supports invasiveness. Notably, there was no evidence for a contribution of EpC to these cld7 activities, i.e. motility and invasiveness promotion likely are genuine cld7 activities.

There remained the question, why the impact of cld7 and EpC on tumor progression appears to be linked. We speculated that cld7 supports EpIC generation, which may contribute to EMT.

### Cld7, EpC and EMT

A cld7^kd^ was accompanied by a minor upregulation in E-cadherin expression and reduced expression of N-cadherin, FN and vimentin. Furthermore, EMT-related transcription factors Snail, Slug, Twist, ZEB1 and TCF4 were downregulated. β-catenin was not enriched in the nucleus and EpIC generation was severely impaired, which features were also seen in HT29 cells after a transient EpC^kd^. On the other hand, EMT gene expression was more pronounced in spheres / holoclones, which express cld7 at a higher level than wt cells. High EpIC acts as a cotranscription factor of β-catenin, FHL2 and Lef-1, the complex initiating, besides others, c-myc, Oct4, Sox2 and Nanog transcription, which was abolished by a γ-secretase inhibitor [19,22]. We observed, as also described before [48-50], that downregulation of EpIC sufficed for a reduction in EMT-related transcription factors Snail, Slug, Twist and TCF4. The same effects being seen in cld7^kd^ and EpC^kd^ cells suggests that the cld7-mediated recruitment of EpC and presenilin2 into GEM facilitates the shift of EpC from a cell-cell adhesion molecule towards a component of EMT.

These data are interpreted in the sense that the metastasis-promoting activity of EpC essentially depends on the association with cld7, which supports EpIC generation.

### Exosome transfer of EMT

CIC are supposed to present a minority of cells that accounts for primary tumor growth as well as for metastasis formation [51]. Furthermore, there is increasing evidence that CIC may fulfill their tasks, at least in part, via exosomes [52]. This was amply demonstrated for preparing a premetastatic niche [53] and was also described for the transfer into non-CIC [54]. As CIC might require a niche [55], but migrating tumor cells are suggested to derive from the rim of the primary tumor [56], we hypothesized that CIC could initiate EMT in non-CIC via exosomes. To obtain a hint towards the likeliness of our hypothesis, holoclones or spheres were taken as CIC-enriched exosome donors and cld7^kd^ cells as poorly metastatic recipients. After coculture, the cld7^kd^ cells changed shape, showed downregulation of E-cadherin, upregulation of N-cadherin and vimentin and gained in motility. The high efficacy of exosomes from spheres or holoclones to induce EMT in poorly metastatic cells requires further elucidation. Nonetheless, taking into account that EMT is transient [38], induction via exosomes could explain the rapid reversion towards MET by dilution / degradation of the initiating signal delivered via exosomes [13,57,58]. This would imply that tumor cells may not require to repeatedly changing their phenotype, but that CIC-derived exosomes suffice for transient EMT.

## CONCLUSION

Cld7 is a CIC marker in CoCa, mostly contributing to tumor progression. This is partly due to the association of p-cld7 with integrins converting them into the activated state that promotes tumor cell motility. More important is the co-operation of cld7 with EpC that allows for the generation of EpIC, a cotranscription factor for β-catenin, which supports EMT transcription factor upregulation. Most notably, spheres and holoclones from cld7-competent cells release exosomes that suffice to transiently induce the EMT phenotype in poorly metastatic cells that exhibit low cld7, but high EpC expression. As the finding implies that the EMT phenotype can be passively and transiently required by the uptake of “CIC”-derived exosomes, it deserves an in depth analysis to decipher whether by a blockade of CIC-derived exosomes the initial step in metastasis can be prevented.

## MATERIAL AND METHODS

### Tumor lines

The CoCa lines Sw948 [32] and HT29 [33] were transfected with cld7 shRNA (Suppl.Tab.1A) using the pSuper/GFP/neo vector. Stable kd lines were established by cloning. For a transient kd of EpC, FlexiTube siRNA Hs_EPCAM_1 was used (Suppl.Tab.1B) (Qiagen, Hilden, Germany). Cells were maintained in RPMI 1640/10%FCS w/wo 0.5μg/ml G418.

### Antibodies and chemicals

see Suppl.Tab.2.

### Tissue preparation and cell isolation

Mice were anesthesized with CO_2_ or were sacrificed by cervical dislocation. Draining lymph nodes (LN), spleen, femora and tibiae, peripheral blood leukocytes (PBL), peritoneal exudate cells (PEC), liver and lung were collected. PEC and bone marrow cells (BMC) were collected by flushing the peritoneal cavity and bones with PBS. PBL were collected by heart puncture. Single cell suspensions were prepared by pressing through fine gauze. Viability (trypan blue exclusion) ranged between 95%-98%.

### Sphere and holoclone selection

Cells (10^3^/ml) were seeded in serum-free RPMI1640 on 0.5% agar precoated 6-well plates. After 1wk half of the medium was exchanged every third day. Spheres were counted after 3wk. Single spheres were picked, dispersed and further passaged. For holoclone selection, 50 cells / cm^2^ were seeded in 3ml RPMI1640/5% FCS in 6 well plates. After 2wk, cells were stained with crystal violet to count the number of holoclones. Alternatively, holoclones were picked and recultured.

### Exosome preparation

Cells were cultured (48h) in serum-free medium. Cleared supernatants (2×10min, 500g, 1×20min, 2000g, 1×30min, 10000g) were centrifuged (90min, 100000g) and washed (PBS, 90min, 100000g). The exosome-containing pellet was resuspended and exosomes were further enriched by sucrose gradient centrifugation. In brief, 1ml exosomes were suspended in 1ml 80% sucrose in HEPES buffer, which was overlaid with 2ml 30% sucrose, 1ml 5% sucrose. After 16h of centrifugation (100000g), 0.4ml fractions were collected. According to the lipid composition of the exosome membrane, exosomes are recovered in the 3^rd^-6^th^ fraction, which corresponds to a density of 1.122-1.171 [34]. Exosomes were resuspended in 50ml PBS and pelleted by centrifugation (90min, 100000g). The pellet was resuspended in a small volume of PBS, filter-sterilized (0.22μm) and stored at −80°C.

### Sucrose density gradient centrifugation

Cell lysates in 2.5M sucrose were overlaid by a continuous sucrose gradient (0.25M-2M) and centrifuged (15h, 150000g), collecting twelve 1ml fractions. Proteins located in glycolipid-enriched membrane subdomains (GEM) are recovered in fraction 2-5.

### Immunoprecipitation (IP), Western blot (WB)

Lysates (30min, 4°C, HEPES buffer, 1% Lubrol or 1% TritonX-100, 1mM PMSF, 1mM NaVO_4_, 10mM NaF, protease inhibitor mix) were centrifuged (13000g, 10min, 4°C), mixed with antibody (1h, 4°C) and incubated with ProteinG-Sepharose (1h). Washed complexes/lysates, dissolved in Laemmli buffer, were resolved on 10%-12% SDS-PAGE. For the recovery of EpIC, Tris-Tricine and 16% SDS-PAGE was used under non-reducing condition. After protein transfer, blocking, blotting with antibodies, blots were developed with ECL.

### Flow-cytometry followed routine procedures

In brief, 1-2.5×10^5^ cells were seeded in 96-well plates. After washing with PBS/1%BSA, cells were incubated with the primary antibody (2-10μg/ml, 40μl, 30min, 4^o^C). Cells were washed 3-times with PBS/1%BSA and incubated with a secondary dye-labeled antibody at predetermined concentration. Negative controls were only incubated with the secondary dye-labeled antibody (40μl, 30min, 4^o^C). For intracellular staining, cells/exosomes were fixed (PBS/1% formaldehyde, 30min, 4^o^C) and permeabilized (PBS/0.5%Tween-20, 20min, 4^o^C). Samples were processed in a FACS-Calibur and evaluated with the CellQuest program.

### Confocal microscopy

Cells on glass-slides were fixed (4% paraformaldehyde, 20min on ice), permeabilized (1% Triton-X100, 4min, on ice), blocked (PBS/1% gelatine, 30min, on ice), incubated with primary antibody (60min, on ice), washed, incubated with fluorochrome-conjugated secondary antibody (60min, on ice), blocked (IgG with irrelevant specificity of the same species as the primary antibody), incubated with a second, dye-labeled primary antibody and washed. Slides were mounted in Elvanol. Digitized images were generated using a Leica LMS780 microscope and the Carl Zeiss Vision software for evaluation. The Z-stack offers 30 positions through the depth of the cell. All pictures were taken at Z-stack 14-16. Depending on the quality of the antibody and the density of marker expression, the intensity for the green channel varied between 700-900 master gain values and for the red channel between 500-750 master gain values. The photosystem automatically generates the single fluorescence and overlay pictures.

### Migration

Cells, in the upper part of a Boyden chamber (RPMI/0.1%BSA), were separated from the lower part (RPMI/20%FCS) by 8μm pore size polycarbonate-membranes. After 24h the lower membrane side was stained (crystal-violet), measuring OD595 after lysis. Migration is presented as % input cells. In an *in vitro* wound healing assay, a subconfluent monolayer was scratched with a pipette tip. Wound closure was controlled by light microscopy.

### Apoptosis

Cells (1×10^5^) were grown for 48h in RPMI/10%FCS containing titrated amounts of cisplatin. Survival was monitored by annexinV/PI staining. In addition, mitochondrial integrity was evaluated by the MTT assay and proliferative activity by ^3^H-thymidine uptake.

### Soft agar assay

Tumor cells in 0.3% agar were seeded on a preformed 1% agar layer counting colonies after 3wk.

### *In vivo* assays

SCID mice received 1×10^6^ tumor cells subcutaneously (sc) or intravenously (iv). Mice were controlled weekly for local tumor growth, short breathing or weight loss. Animals were sacrificed when the local tumor reached 1.5cm diameter, mice lost >10% weight or latest after 210d. Animals were bled by heart puncture, all hematopoietic organs, as well as liver and lung were excised and single cell suspensions were obtained by meshing through fine gauze. Cell suspensions were subjected to flow cytometry evaluating the percent of EpC^high^ and Tspan8^+^ cells. Remaining cells were cultured in RPMI1640/10%FCS for several weeks. The non-transformed cells die, but mostly dormant tumor cells start to form colonies after several weeks. Animal experiments were Government-approved (Baden-Wuerttemberg, Germany).

### Statistics

P values <0.05 (two-tailed Student's t-test, Kruskal-Wallis test) were considered significant.

### Authorship

RP, SH, WM, FT and MZ performed and analyzed experiments, MWB helped with writing the manuscript, FT and MZ planned and analyzed experiments and wrote the manuscript.

## SUPPLEMENTARY MATERIAL, FIGURES AND TABLES



## Abbreviations

BM: bone marrow
BMC: BM cells
CIC: cancer initiating cells
cld7: claudin-7
CoCa: colon cancer
EMT: epithelial mesenchymal transition
EpC: EpCAM
EpIC: EpCAM intracellular domain
GEM: glycolipid-enriched membrane domains
IP: immunoprecipitation
iv: intravenous
kd: knockdown
ko: knockout
LN: lymph node
LNC: LN cells
PBL: peripheral blood leukocytes
PEC: peritoneal exudate cells
SC: spleen cells
sc: subcutaneous
TB: tumor-bearer
WB: Western blot
wt: wild type
