# Supplementary material for: Claudin-7 promotes the epithelial – mesenchymal transition in human colorectal cancer
Source: Oncotarget. 2014 Dec 3;6(4):2046–63. doi: 10.18632/oncotarget.2858 (PMC4385835; doi:10.18632/oncotarget.2858)
Supplement: Supplementary file 1 [file oncotarget-06-2046-s001.pdf]

Claudin-7 promotes the epithelial – mesenchymal transition in human colorectal cancer

Supplementary Material

Suppl. Table 1 **shRNA and siRNA**
Suppl.Table 1A **Cld7 siRNA and shRNA vector insert**

siRNA humCldn7-4
target sense: TCGGACCTACAGCCCAGGATA
target antisense: TATCCTGGGCTGTAGGTCCGA

vector insert
5´-
GATCCCC**TCGGACCTACAGCCCAGGATA**TTCAAGAGA**TATCCTGGGCTGTAGGTCCG**
ATTTTTA-3`
3´-
GGG**AGCCTGGATGTCGGGTCCTAT**AAGTTCTCT**ATAGGACCCGACATCCAGGCT**AAA
AATTCGA-5´

Suppl. Table 1B **siRNA Hs_TACSTD1_6**

target sequence: 5´-AACTATATAATTTGAAGATTA-3`
sense strand: 5´-CUAUAUAAUUUGAAGAUUATT-3`
Antisense strand: 5´-UAAUCUUCAAAUUAUAUAGTT-3`

Suppl. Table 2 **Chemicals and Antibodies**
Suppl. Table 2A **Chemicals**

substance	dose	supplier
AnnexinV-FITC / -APC	variable	Becton Dickinson, HD, G
Cisplatin	1-30µg/ml	Sigma, Munich, G
Methyl-b-cyclodextrin	20mM, 30min	Merck, Darmstadt, G
Matrigel	invasion: 1:5	Becton Dickinson, HD, G
Phalloidin	0.5µg/ml	Becton Dickinson, HD, G
PI	variable	Becton Dickinson, HD, G
PMA	10^{-8}M	Sigma Munich, G

Suppl. Table 2B **Antibodies**

Antibody	origin	supplier
Actin	mouse	Becton Dickinson, HD, G
BAD	mouse	Becton Dickinson, HD, G
BAX	mouse	Becton Dickinson, HD, G
β-catenin	rabbit	Becton Dickinson, HD, G
Bcl2	mouse	Becton Dickinson, HD, G
BclXl	mouse	Becton Dickinson, HD, G
Caspase3	mouse	Becton Dickinson, HD, G
act.Caspase3	rabbit	Becton Dickinson, HD, G
Caspase8	rabbit	Becton Dickinson, HD, G
Caspase9	mouse	Becton Dickinson, HD, G
cleav.Casp.9	rabbit	Cell Signalling, Frankfurt , Germany
CD13	mouse	Becton Dickinson, HD, G
CD26	mouse	ImmunoTools, Friesoythe, G
CD29	mouse	Becton Dickinson, HD, G
CD44 (25-32)	mouse	ref [1]
CD44v6 (vFF18)	mouse	ref [2]
CD49c	mouse	Becton Dickinson, HD, G
CD49f	mouse	Becton Dickinson, HD, G
CD104	rabbit	Becton Dickinson, HD, G
CD133	rabbit	Becton Dickinson, HD, G
CD166	mouse	Becton Dickinson, HD, G
CD184	mouse	Becton Dickinson, HD, G
cld7	guinea pig	ref [3]
E-cadherin	mouse	Becton Dickinson, HD, G
EGFR	rabbit	AnaSpec, San Jose, Ca, US
EpCAM (HEA125)	mouse	ref [4]
EpIC	rabbit	home made, unpublished
FGF	mouse	Becton Dickinson, HD, G
FN	mouse	Becton Dickinson, HD, G
Ki-67	mouse	Becton Dickinson, HD, G
MDR	mouse	Biolegend, San Diego, Ca, US
MET	mouse	Cell Signaling, Frankfurt, G
MMP2	rabbit	Dianova, Hamburg, G
MMP3	rabbit	Santa Cruz, HD, G
MMP7	rabbit	Santa Cruz, HD, G
MMP9	rabbit	Dianova, Hamburg, G
MMP13	rabbit	Dianova, Hamburg, G
MMP14	rabbit	Santa Cruz, HD, G
N-Cadherin	mouse	Becton Dickinson, HD, G

Suppl.Table2B continued

Antibody	origin	supplier
Notch	mouse	Biolegend, San Diego, Ca, US
p-Akt	mouse	Becton Dickinson, HD, G
p-BAD	mouse	Cell Signaling, Frankfurt, G
p-β-catenin	rabbit	BioTrend, Cologne, G
p-cld7	rabbit	Sigma, Munich, G
p-PI3K	rabbit	Cell Signaling, Leiden, NL
presenilin	rabbit	Santa Cruz, HD, G
slug	rabbit	Santa Cruz, HD, G
snail	rabbit	Santa Cruz, HD, G
TACE	rabbit	Santa Cruz, HD, G
TCF4	mouse	Santa Cruz, HD, G
TGFβ	mouse	Becton Dickinson, HD, G
Tspan8 (CO029)	mouse	ref [5]
twist	rabbit	Becton Dickinson, HD, G
uPAR	mouse	Calbiochem, Darmstadt, G
vimentin	mouse	Becton Dickinson, HD, G
ZEB-1	rabbit	Santa Cruz, Heidelberg, G
dye or biotin labeled secondary antibodies / Streptavidin		Dianova, Becton Dickinson, Amersham

1. Zuckermann FA, Binns RM, Husmann R, Yang H, Carr MM, Kim YB, Davis WC, Misfeldt M, Lunney JK. Analysis of monoclonal antibodies reactive with porcine CD44 and CD45. Vet Immunol Immunopathol. 1993; 43: 293-305.
2. Seiter S, Tilgen W, Herrmann K, Schadendorf D, Patzelt E, Möller P, Zöller M. Expression of CD44 splice variants in human skin and epidermal tumours. Virchows Arch. 1996; 428: 141-149.
3. Ladwein M, Pape UF, Schmidt DS, Schnölzer M, Fiedler S, Langbein L, Franke WW, Moldenhauer G, Zöller M. The cell-cell adhesion molecule EpCAM interacts directly with the tight junction protein claudin-7. Exp Cell Res. 2005; 309: 345-357.
4. Momburg F, Moldenhauer G, Hämmerling GJ, Möller P. Immunohistochemical study of the expression of a Mr 34,000 human epithelium-specific surface glycoprotein in normal and malignant tissues. Cancer Res. 1987; 47: 2883-2891.
5. Sela BA, Steplewski Z, Koprowski H. Colon carcinoma-associated glycoproteins recognized by monoclonal antibodies CO-029 and GA22-2. Hybridoma. 1989; 8: 481-491.

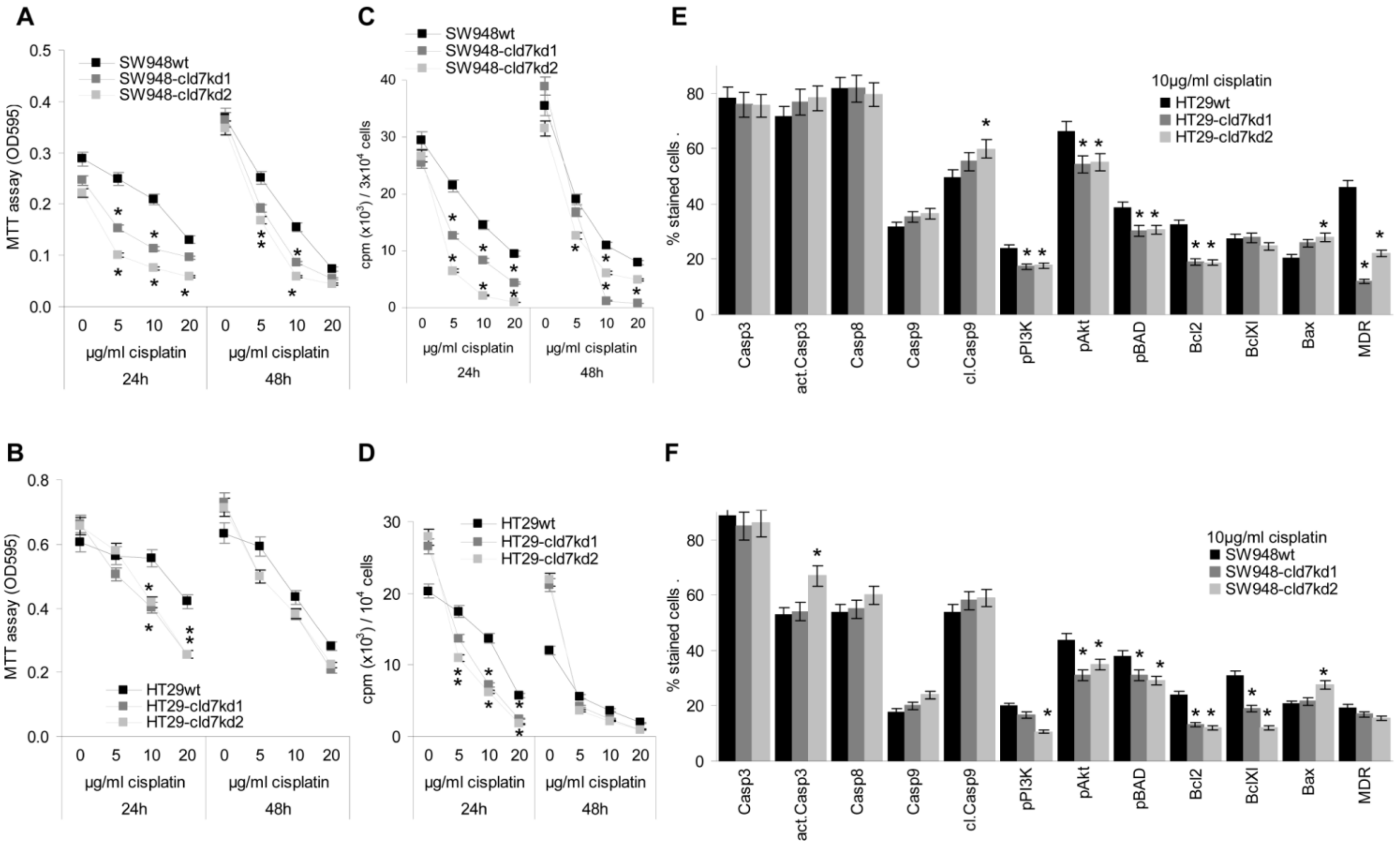

Suppl.Figure 1: Apoptosis resistance and pro- and anti-apoptotic protein expression in $HT29^{wt}$ and $SW948^{wt}$ and -$cld7^{kd}$ cells: (A-D) $SW948^{wt}$ and $HT29^{wt}$ and -$cld7^{kd}$ cells were cultured for 24h – 48h in the presence of titrated amounts of cisplatin. (A,B) Mitochondrial integrity was evaluated by the MTT assay; the mean±SD OD595 (triplicates) is shown; (C,D) proliferative activity was evaluated by ^{3}H-thymidine uptake, mean cpm±SD (triplicates) is shown. (E,F) Caspase activity and expression of pro- and anti-apoptotic molecules was evaluated by flow cytometry in cisplatin-treated $SW948^{wt}$ and $HT29^{wt}$ and -$cld7^{kd}$ cells by flow cytometry (mean±SD, 3 assays); (A-F) significant differences between wt and $cld7^{kd}$ cells: *. Impaired apoptosis resistance of $cld7^{kd}$ cells is accompanied by reduced activation of the PI3K/Akt pathway.

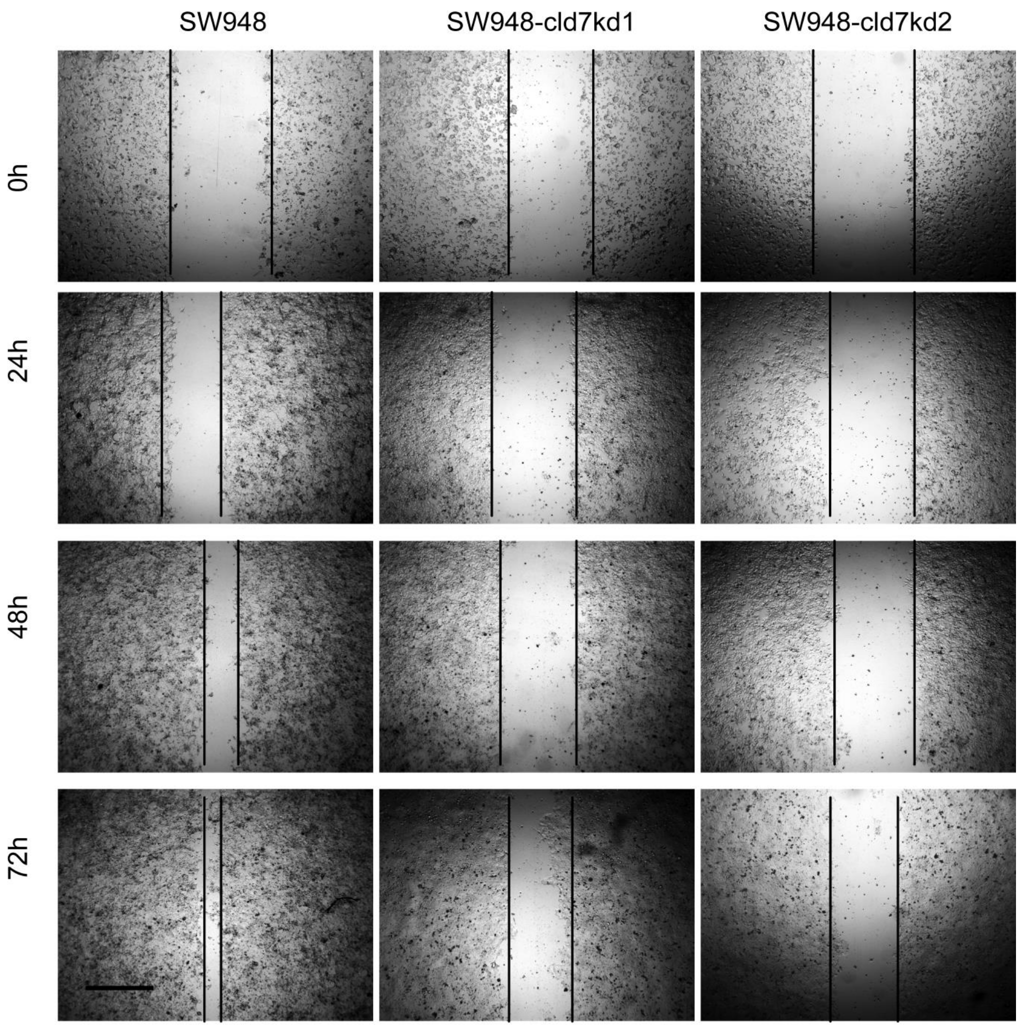

Suppl.Figure 2: <u>The impact of cld7 on SW948 cell migration:</u> SW948^{wt} and -cld7^{kd} cells were seeded in 24-well plates. When reaching subconfluence, the monolayer was scratched with a yellow pipette tip; wound healing was followed for 72h; representative examples (scale bar: 250µm) are shown. Wound healing is retarded in SW948-cld7^{kd} cells.

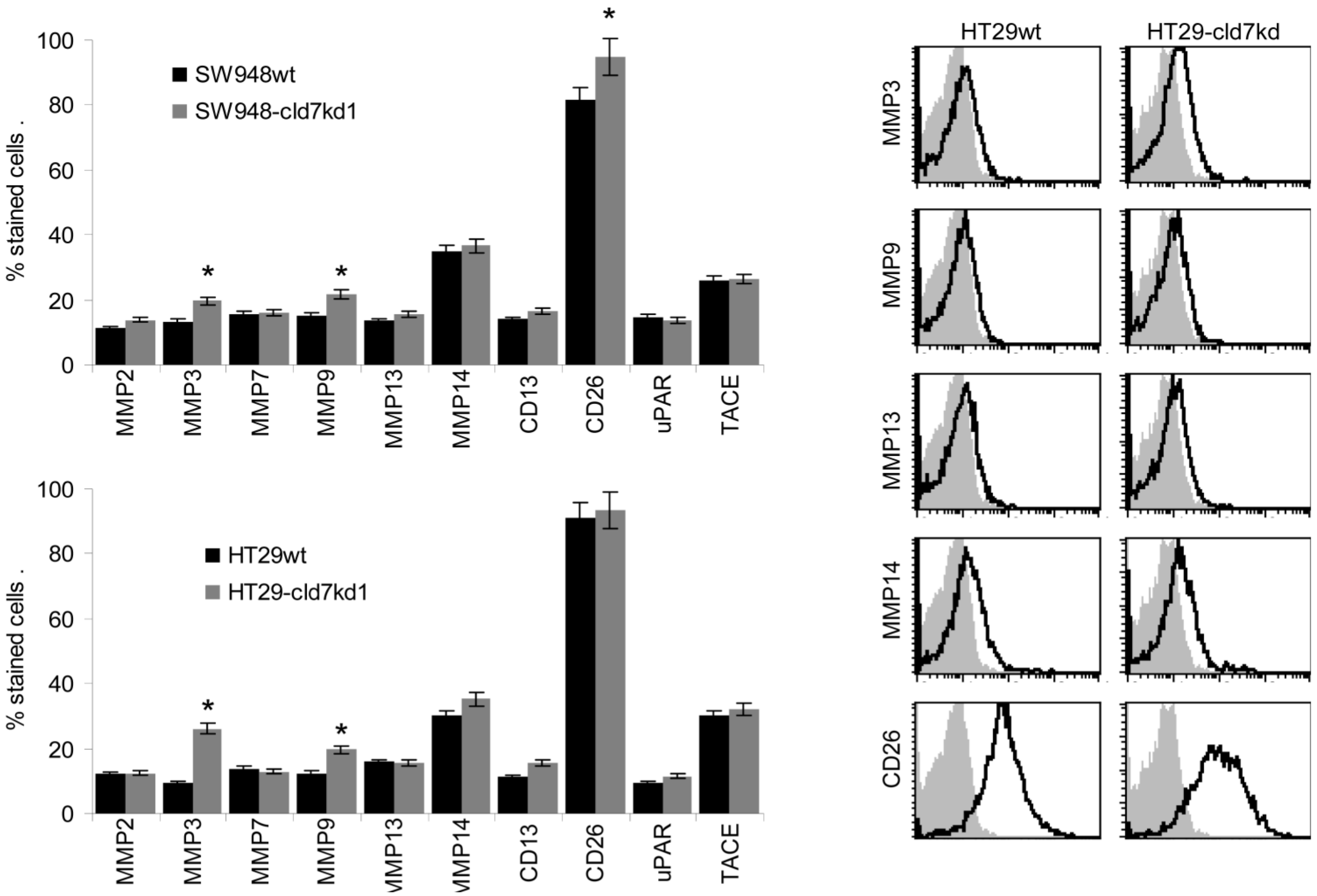

Suppl.Figure3: <u>The impact of cld7 on protease expression in colon cancer lines</u>: Expression of MMPs, uPAR and the dipeptidases CD13 and CD26 was evaluated by flow cytometry. The mean % stained cells±SD (3 assays) and representative examples are shown; significant differences between $HT29^{wt}$ and $SW948^{wt}$ versus $-cld7^{kd}$ cells: *.

Reduced cld7 expression is accompanied by a slight upregulation of MMP3, MMP9 and MMP14 (only HT29). Expression of other proteases is not affected.

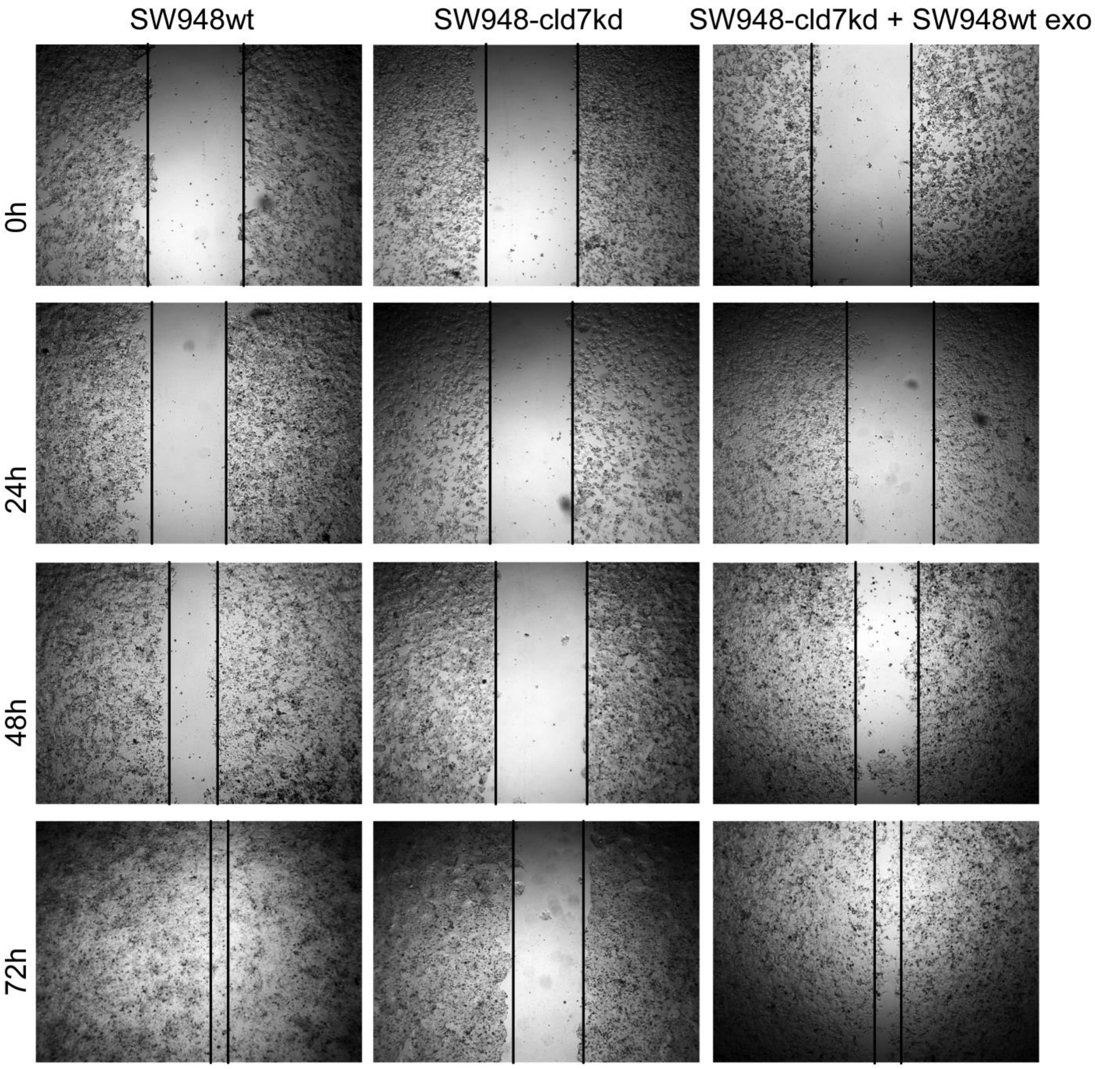

Suppl.Figure 4: Exosome-promoted migration of SW948-cld7^{kd} cells: SW948-cld7^{kd} cells were seeded in 24w plates; subconfluent cultures were scratched with a pipette tip. Wound healing was observed for 72h in the presence of medium with exosome-depleted FCS or with exosomes from wt, cld7^{kd} or sphere-derived cells; representative examples (scale bar: 250µm).
